# Multiepitope Dendrimeric Antigen-Silica Particle Composites as Nano-Based Platforms for Specific Recognition of IgEs

**DOI:** 10.3389/fimmu.2021.750109

**Published:** 2021-12-03

**Authors:** Violeta Gil-Ocaña, Isabel M. Jimenez, Cristobalina Mayorga, Inmaculada Doña, Jose Antonio Céspedes, Maria I. Montañez, Yolanda Vida, Maria J. Torres, Ezequiel Perez-Inestrosa

**Affiliations:** ^1^ Universidad de Málaga-Instituto de Investigación Biomédica de Málaga (IBIMA), Dpto. Química Orgánica, Campus de Teatinos s/n, Málaga, Spain; ^2^ Centro Andaluz de Nanomedicina y Biotecnología-BIONAND, Parque Tecnológico de Andalucía, Málaga, Spain; ^3^ Allergy Research Group, Instituto de Investigación Biomédica de Málaga-Instituto de Investigación Biomédica de Málaga (IBIMA), Málaga, Spain; ^4^ Allergy Unit, Hospital Regional Universitario de Málaga, Málaga, Spain; ^5^ Universidad de Málaga-Instituto de Investigación Biomédica de Málaga (IBIMA), Dpto. Medicina, Campus de Teatinos s/n, Málaga, Spain

**Keywords:** dendrimeric antigen, silica nanoparticles, biepitope nanocomposites, diagnostic test, drug allergy, penicillins, specific IgE

## Abstract

β-lactam antibiotics (BLs) are the drugs most frequently involved in drug hypersensitivity reactions. However, current *in vitro* diagnostic tests have limited sensitivity, partly due to a poor understanding of *in vivo* drug–protein conjugates that both induce the reactions and are immunologically recognized. Dendrimeric Antigen-Silica particle composites (DeAn@SiO_2_), consisting on nanoparticles decorated with BL-DeAns are promising candidates for improving the *in vitro* clinical diagnostic practice. In this nano-inspired system biology, the synthetic dendrimer plays the role of the natural carrier protein, emulating its haptenation by drugs and amplifying the multivalence. Herein, we present the design and synthesis of new multivalent mono- and bi-epitope DeAn@SiO_2_, using amoxicillin and/or benzylpenicillin allergenic determinants as ligands. The homogeneous composition of nanoparticles provides high reproducibility and quality, which is critical for *in vitro* applications. The suitable functionalization of nanoparticles allows the anchoring of DeAn, minimizing the nonspecific interactions and facilitating the effective exposure to specific IgE; while the larger interaction area increments the likelihood of capturing specific IgE. This achievement is particularly important for improving sensitivity of current immunoassays since IgE levels in BL allergic patients are very low. Our data suggest that these new nano-based platforms provide a suitable tool for testing IgE recognition to more than one BL simultaneously. Immunochemical studies evidence that mono and bi-epitope DeAn@SiO_2_ composites could potentially allow the diagnosis of patients allergic to any of these drugs with a single test. These organic–inorganic hybrid materials represent the basis for the development of a single screening for BL-allergies.

## 1 Introduction

Nanotechnology broke into society a few decades ago and is currently present in many aspects of our lives. Its great success is due to its proposal to provide small solutions to big problems. Nanotechnology encompasses several fields of science as chemistry, physics, mathematics, pharmacy, and computer or environmental sciences, being able to create different materials and devices with a huge range of applications. Furthermore, the possibility of combination between a nanosized material into hybrid nanostructures, controlling its size, shape and composition, gives the scientific community a powerful tool to design nanomaterials with multiple functionalities, which has a special importance in biomedicine (1). Nanoparticles are particularly important for biomedical purposes, as their unique properties like high surface-to-volume ratio, similar size of biomolecules, chemically tunable surface or, in many cases, particular physicochemical properties make them unique platforms for certain applications. The huge potential of nanoparticles in the field of nanomedicine has led the scientific community to pay more and more attention to this kind of nanomaterials, which have been very useful in both *in vivo* and *in vitro* applications (2). Focusing on the *in vitro* applications, the use of nanoparticles in diagnosis tends to lead to better assays sensitivity and selectivity, assuming a substantial improvement over conventional diagnostic systems (3). Silica (SiO_2_)-composite nanoparticles are undoubtedly the most widely used for a wide variety of applications, being extensively used for diagnostic purposes (4). Their synthesis is cheap and easy, as particle size can be controlled simply, is biocompatible, and allows a versatile surface chemical modification. They also provide a robust solid support for the immobilization of different biomolecules, which makes them ideal platforms in immunoassay applications (5). These highly selective and sensitive assays take the advantage of the specific interaction between an antigen and an antibody, and constitute a powerful tool to determine diverse biological substances.

Herein we focus on the development of nanoparticles for immunoassays applications in the field of the diagnosis of allergic reactions to drugs. Betalactam antibiotics (BLs) are drugs especially important as, due to their extensive use against bacterial infections, they are the most commonly involved in hypersensitivity reactions, becoming a worldwide problem (6). BL allergy can be induced by different immunological mechanisms, being mostly induced by IgE mediated mechanisms (7). Symptoms can range from simple skin problems to anaphylactic shock (6). Their diagnosis is complex, involving the clinical history and *in vivo* methods (skin test or drug provocation tests) which can be risky. Moreover, *in vitro* tests consisting on the basophil activation test and the serum specific IgE (sIgE) determination are helpful for diagnosis although with lower sensitivity (8). Based on patient safety, the use of these complementary *in vitro* methods in combination with skin tests (STs) is preferred for avoiding the performance of the drug provocation tests (9, 10).

Among *in vitro* tests, serum drug sIgE determination using immunoassays has traditionally been the most common *in vitro* method for the diagnosis of BL allergy (11). Nowadays the most widely applied method in clinical diagnosis is the commercial ImmunoCAP (Thermo-Fisher), a standardized fluoro-enzyme immunoassay (FEIA). However, when applied to BL allergy, sensitivity is low (0-50%) and variable (10, 12, 13). Moreover, false benzylpenicillin (BP) allergy diagnoses have been reported with ImmunoCAP (14, 15). The previous alternative immunoassay for quantification of BL-sIgE consisted on the conventional radioallergosorbent test (RAST), which is nowadays only performed in specialized laboratories for research purposes (11). These customized immunoassays show higher sensitivity (43-75%) and specificity (68-83%) for BLs (8). The main difference between them refers to the antibody detection technique (fluorescence *vs* radioactivity), although the cellulose materials in solid phase (sponge matrix contained in a small capsule *vs* paper discs) are also used for capturing the target sIgE (16). Both solid phases of the commercial ImmunoCAP or the conventional RAST consist on a cellulose polymer activated with cyanogen bromide where the drug-poly-L-lysine (PLL) conjugates are covalently attached. On the other hand, the configuration of the home-made RAST is much more versatile, allowing customizing carriers, solid phases, and activation chemistry (17).

A critical point in this technology is the nature of the drug-carrier conjugate, based on the hapten hypothesis, which states that a drug can only induce an immediate allergic reaction after covalent binding to a carrier protein (18), which is specifically recognized by sIgE antibodies. However, the poor understanding of *in vivo* drug–protein conjugates could explain the suboptimal diagnostic value of both methodologies. Besides the structure adopted by the drug after covalently binding to the protein and the nature of the carrier molecule, other factors as the solid phase material, its chemical functionalization and sIgE detection techniques can also influence the sensitivity and specificity of immunoassays (8, 11, 17).

The chemical nature of the hapten(drug)-carrier(protein) conjugate has been extensively studied with the aim to emulate *in vitro* the *in vivo* recognition process (18, 19). Moreover, numerous attempts to optimize and increase the sensitivity of the homemade RAST assays have been addressed taking into account the promising benefits of nanomaterials (20, 21). In this context, PAMAM dendrimers have been proposed as perfect substitutes of the carrier protein in the hapten-carrier conjugate. These dendrimer structures provide structural homogeneity, integrity, and controlled multivalence, which allows a high density of haptens in their surface in a reproducible synthetic way (22). These dendrimeric antigen conjugates (DeAns) showed sIgE recognition toward the drug (BP or amoxicillin (AX)) anchored on its periphery (22, 23). Their attachment to cellulose discs allowed the fabrication of discs in which the degree of functionalization, the chemical structure of the conjugate and the linkers were perfectly determined (24). Both the greater degree of functionalization of the cellulose surface disc (24, 25) and the higher generation of dendrimer with which the cellulose was grafted involved an increased density of haptens in the material (26). These factors demonstrated to influence the ability of such materials to capture sIgE from patient sera, showing potential for their use in drug allergy diagnostic tests. These dendrimers, as new carrier molecules, and chemistry linkages, can be combined with other solid supports: nano-sized zeolites (25) and silica particles composites (27) as their high surface area could translate into an improved sensitivity. *In vitro* evaluation of resulting materials with sera from BL allergic patients led to a successful proof of concept. However, preparation and functionalization of such zeolite turned out to be too expensive to standardize its use for clinical practice, whereas advantages of silica particles justify their use as solid phase for obtaining DeAn-inorganic-organic composites that provide highly specific and sensitive tests for detection of AX-sIgE (27).

Complex and intrinsic issues for diagnosing BL-allergic patients are the fact that drug-sIgE levels are very low (affecting to the low sensibility of immunossays) and the possibility of presenting reactions to more than one kind of BL (affecting to specificity). Based on their sIgE recognition, patients can be classified as allergic to only one kind of penicillin or to several penicillins, or can also present cross-reactivity with other BLs containing similar side chains (28). Diagnosis of such patients implies carrying out the diagnostic algorithm for each suspected penicillin, is time consuming, and requires great efforts in the clinical routine. Therefore, tools for a single screening of BL-IgE antibodies with different specificities could be of interest for a quick diagnosis. In this context, well-characterized bi-epitope DeAns,decorated with both AX and BP allergenic determinants [named amoxicilloyl (AXO) and benzylpenicilloyl (BPO)], showed the ability of specific recognition by IgE of patients allergic to both, AX and BP, with potential in the development for universal *in vitro* detection of BL allergies (23).

Based on those promising results, herein we present the preparation of new multivalent mono and bi-epitope DeAn@SiO_2_ composites, consisting on silica nanoparticles decorated with BL-DeAns. PAMAM-G2 dendrimers were selected as carrier molecule and AX and/or BP as the allergenic determinants. The chemical modification of the particle surface was designed in order to allow checking the quality and composition of the nanocomposites, using standard techniques as zeta potential or IR measurements and NMR spectroscopy. The controlled homogeneous composition of the nano-composites provides the high reproducibility needed for their *in vitro* applications. The methodology used to anchor the DeAns was optimized in order to minimize the nonspecific interactions and facilitate the effective exposure of the hapten-carrier molecules to specific IgE. This, together with the larger interaction area provided by the nanoparticles, increments the likelihood of capturing sIgE, which is particularly important for overcoming the low sensitivity of current immunoassays. Their ability to quantify AX-sIgE and BP-sIgE was evaluated in sera patients and compared with current immunoassays techniques, showing improvements in terms of sensibility and specificity.

## 2 Materials and Methods

### 2.1 Nanoparticle Chemical Studies

Standard chemicals were obtained from Aldrich or VWR and used without further purification. PAMAM dendrimers were purchased from Aldrich. Milli-Q™ water was obtained by using the ultrapure, Millipore^®^ Direct-Q^®^ 3. Phosphate buffer saline (PBS) and 4M Lithium acetate buffer pH 5.3 was prepared as described elsewhere (29, 30).

#### 2.1.1 SiO_2_ Particles Synthesis

Silica particles were prepared according to a previously reported procedure (31). 7.5 mL of ammonium hydroxide were added to 55 mL of absolute ethanol and the mixture was stirred for 15 minutes. Then, tetraethyl orthosilicate (TEOS, 4.5 mL) was added to obtain the silica microspheres. The particles were centrifuged and washed with HCl 1M and Milli-Q™ water. Finally, 1 g of silica particles (SiO_2_) was obtained.

#### 2.1.2 SiO2 Particles Surface Modification

##### 2.1.2.1 Amino Functionalization

Prepared particles (SiO_2_, 1 g) were suspended in 300 mL of toluene and sonicated for 15 min. Then, 3-(aminopropyl)-triethoxysilane (APTES, 1 mL) was added and mixture was sonicated for 1 hour. The solution was refluxed under stirring overnight after the addition of 1 mL extra of APTES. The obtained particles were washed with toluene (x 3 times) and Milli-Q™ water (x 3 times) and finally lyophilized to afford the desired amino functionalized particles (SiO_2_-NH_2_).

##### 2.1.2.2 Carboxyl Functionalization

Amino functionalized particles (SiO_2_-NH_2_, 900 mg) were suspended in dimethylformamide (DMF, 33mL) and sonicated for 15 min. Then, succinic anhydride (2.30 g) in 17 mL of DMF were added to the previous mixture and sonicated for additional 15 min. Triethylamine (TEA, 286 µL) were then added and the solution stirred at room temperature overnight. The particles were washed with DMF (x 3 times) and Milli-Q™ water (x 3 times) and finally lyophilized to afford the desired carboxyl functionalized particles (SiO_2_-CO_2_H).

##### 2.1.2.3 Dendrimer Functionalization

Carboxyl functionalized particles (SiO_2_-CO_2_H, 800 mg), 1-(3-(dimethylamino) propyl)-3-ethylcarbodiimide hydrochloride (EDCI, 276 mg) and *N*-hydroxisuccinimide (NHS, 320 mg) were suspended in 13 mL of anhydrous DMF and sonicated for 15 min. To this solution, 12 mL of anhydrous DMF containing PAMAM-G2 dendrimer (137 mg) were added. The resulting suspension was sonicated for 15 min and stirred at room temperature overnight. The particles were washed with DMF (x 3 times) and Milli-Q™ water (x 3 times) and finally lyophilized to afford the desired dendrimer functionalized particles (SiO_2_-De).

##### 2.1.2.4 β-Lactam Antibiotics (BLs) Functionalization

Dendrimeric particles (SiO_2_-De, 150 mg) were suspended in 4 mL of a freshly prepared solution of the corresponding BL (10 mg/mL of AX, BP or a 1:1 mixture of both) in 0.05 M Na_2_CO_3_/NaHCO_3_ aqueous buffer at pH 10.2. The suspension was then sonicated for 15 min and stirred at 4°C for 7 days. During this period, 10 mg of the corresponding BL were added at approximately 24 h intervals. The particles were washed with PBS buffer (x 3 times) and Milli-Q™ water (x 3 times) and finally lyophilized to afford the desired dendrimeric antigen-silica particle composites (DeAn@SiO_2_). These include DeAXO@SiO_2_, DeBPO@SiO_2_, and DeAXO-BPO@SiO_2_, respectively.

#### 2.1.3 Silica Particles Characterization

##### 2.1.3.1 Transmission Electron Microscopy (TEM)

Transmission electron microscopy (TEM) images (PHILIPS CM-200 instrument) were used to analyze particle size and morphology. Samples were prepared by suspending 1mg of the corresponding particles in 1 mL of Milli-Q™ water.

##### 2.1.3.2 Dynamics Light Scattering (DLS)

A Malvern Zetasizer Nano ZS90 instrument was used to determine the hydrodynamic diameter and stability of the particles in aqueous solutions. The instrument was equipped with a “red laser” (λ= 633 nm) and measurements were carried out with a detection angle of 90°. Samples were prepared by suspending 1 mg of the corresponding particles in 3 mL of Milli-Q™ water.

##### 2.1.3.3 Z Potential Measurements

Z Potential measurements were carried out using a Malvern Zetasizer Nano ZS90 instrument. Samples were prepared by suspending 1 mg of the corresponding particles in 3 mL of phosphate buffer saline (PBS). Measurements were done in duplicate.

##### 2.1.3.4 Fourier Transformed Infrared (FTIR)

A Nicolet Nexus spectrometer (Thermo Fisher Scientific) equipped with a Smart Golden Gate attenuated total reflectance (ATR) accessory was used. Measurements were carried out with 5 mg of the corresponding particles.

##### 2.1.3.5 Quantification of Free Primary Amino Groups

The quantification of free primary amino groups present on the particle surface was carried out by using a previously described procedure (25, 26, 30). The ninhydrin test solution was prepared dissolving ninhydrin (300 mg) and hydrindantin (45 mg) in water-free DMSO (11.25 mL). Then, 4 M Lithium acetate buffer pH 5.3 (3.75 mL) was added and the solution was deoxygenated. The corresponding particles (1 mL of a 3mg/mL Milli-Q™ water suspension) were placed in a sealed tube and 1 mL of ninhydrin solution was then added. The solution was then heated to 100°C for 15 minutes. Then, 5mL of 50% water/EtOH was added. The reaction mixture was filtered and the absorbance (λ=570nm) was measured with a VARIAN CARY 100BIO UV-Visible spectrophotometer. A calibration was performed with aqueous *n*-butylamine solutions in a concentration range of 100-650 µM. The measurements were carried out in triplicate, resulting in reproducible values.

##### 2.1.3.6 ^1^H-NMR Spectra of DeAn@SiO_2_

^1^H-NMR spectra were acquired on a Bruker Avance 600 MHz spectrometer equipped with a 4mm TXI HR-MAS probe, using the zgpr sequence (*viz*. a low-power presaturation pulse followed by a 90° high-power pulse). A total of 32k data points were obtained in 64 scans at 25°C at a spinning rate of 5 kHz. Samples were prepared by suspending 20 mg of DeAn@SiO_2_ in 600 µL of D_2_O. The suspension was transferred to a HR-MAS zirconium rotor. DSL measurements, TEM images, estimation of the degree of functionalization of the particles based on the quantification of free primary amino groups and NMR spectra are available in the **Supplementary Material**.

### 2.2 Patients and *In Vivo/In Vitro* Diagnosis Methods

#### 2.2.1 Patients Selection

The studied group of subjects was selected from patients who came to the Allergy Service of the Regional University Hospital of Málaga. The study was conducted according to the Declaration of Helsinki principles and was approved by the Provincial Ethics Committee of Malaga. All subjects included in the study were informed orally and signed the corresponding informed consent.

Patients diagnosed of an immediate allergic reaction to AX and tolerant subjects to BLs, according to the European Academy of Allergy and Clinical Immunology (EAACI) guidelines (9, 32), were eligible for the study. A total of 21 patients with confirmed AX allergy were chosen after an allergological workup including clinical history of reaction after AX intake and positive ST to AX (**Table 1**). A requirement for the inclusion of patients was the presence of AX-sIgE determined by ImmunoCAP.

**Table 1:** Clinical data and classification (based on skin test and ImmunoCAP results) of patients diagnosed with an immediate allergic reaction to AX included in the study.

Pat	Age	Sex	Reaction	Grade Severity^a^	Drug involved in the reaction	INT (days)	Skin test		ImmunoCAP	
							BP^b^	AX	BP	AX
1A	36	F	Anaphylaxis	II	AX-CLV	201	–	+	–	+
2A	57	F	Anaphylaxis	II	AX	157	–	+	–	+
3A	66	F	Anaphylaxis	II	AX/Nolotil, Ibuprofen	143	–	+	–	+
4A	58	F	Urticaria/AE	I	AX/Aztreonam	8280	–	+	+	+
5A	19	M	Anaphylaxis	II	AX-CLV	4288	–	+	+	+
6A	57	F	Anaphylactic shock	III	AX-CLV	265	–	+	+	+
7A	50	M	Anaphylaxis	II	AX-CLV	371	ND	+	–	+
8A	32	F	Anaphylaxis	II	AX-CLV	1038	ND	+	–	+
9A	44	F	Urticaria/AE	I	AX-CLV	2137	ND	+	–	+
10A	15	F	Urticaria/AE	I	AX-CLV/AX	205	ND	+	–	+
11A	16	F	Urticaria/AE	I	AX	206	ND	+	–	+
12A	49	F	Anaphylactic shock	III	AX-CLV/AX	177	ND	+	–	+
13B	55	M	Urticaria/AE	I	AX-CLV	374	ND	+	+	+
14B	30	M	Anaphylactic shock	III	AX	44	ND	+	+	+
15B	31	F	Anaphylactic shock	III	AX	45	ND	+	+	+
16B	32	M	Anaphylactic shock	III	AX	46	ND	+	+	+
17B	59	M	Anaphylaxis	II	AX-CLV	79	+	+	+	+
18B	46	M	Urticaria/AE	I	AX	315	+	+	+	+
19B	55	F	Anaphylaxis	II	AX-CLV	93	+	+	+	+
20B	58	M	Urticaria/AE	I	BP	5792	+	+	+	+
21B	58	F	Anaphylactic shock	III	AX	138	+	+	+	+

Pat, Patient; F, female; M, male; AE, angioedema; INT, time interval between reaction and study; AX, amoxicillin; CLV, clavulanic acid; BP, benzylpenicillin; (+), positive; (-): negative; ND, not determined.

a Grading system for generalized hypersensitivity reactions: I Mild (skin and subcutaneous issues only); II Moderate (features suggesting respiratory, cardiovascular, or gastrointestinal involvement); III Severe (hypoxia, hypotension, or neurologic compromise) (Brown SG. J Allergy Clin Immunol. 2004 Aug;114(2):371-6).

b BP reagents include BP-OL, benzylpenicilloyl-octalysine and MD, minor determinant.

Patients were classified as AX selective reactors with tolerance to BP (Group A) when they had negative results in ST to BP determinants or BP-sIgE quantified by ImmunoCAP. Patients were classified as cross-reactors to penicillins (BP and AX) (Group B) when they had positive results in ST to BP determinants or BP-sIgE determined by ImmunoCAP.

Ten subjects with confirmed tolerance to penicillins who also showed undetectable levels of BP-sIgE and AX-sIgE according to ImmunoCAP results, were included as negative controls (**Table S1**). Four of these subjects showed high levels of total IgE.

#### 2.2.2 Skin Test

Skin prick tests and, if negative, intradermal tests were performed as described (9, 32), using AX (Diater laboratories, Madrid, Spain) at 20 mg/mL and BP determinants (33) from DAP Penicillin^®^ test Kit (Diater laboratories, Madrid, Spain). This kit includes the following reagents: benzylpenicilloyl-octa-L-lysine (BP-OL) used at 0.04 mg/mL (8.64·10^–5^ M concentration of the benzylpenicilloyl (BPO) moiety), and the minor determinant (MD) at 0.5 mg/mL (1.5·10^–3^ M concentration of sodium benzylpenilloate). Readings were done after 20 min and considered positive if a wheal surrounded by erythema appeared following criteria previously described (34).

#### 2.2.3 *In Vitro* sIgE Determination

All sIgE determinations were performed *in vitro* using sera from allergic and tolerant subjects and the following commercial or customized materials.

##### 2.2.3.1 ImmunoCAP

The FEIA ImmunoCAP (Thermo-Fisher) was used for detecting serum BP-sIgE (with allergen c1, penicilloyl G) and AX-sIgE (with allergen c6, amoxicilloyl) and serum total IgE in patients and controls following manufacturer´s instructions. Results of drug-sIgE were considered positive according to the cutoff ≥ 0.1 kU A/L.

##### 2.2.3.2 RAST Using PLL-Discs

The *in vitro* IgE determination by RAST was performed adapting conventional methodology, using the drug of interest (BP or AX) conjugated to poly-L-lysine (PLL) over the cellulose disc solid support (35).

###### 2.2.3.2.1 Preparation of Discs

Cellulose discs (6 mm in diameter) were cut from Whatman 54 filter paper. These cellulose discs were activated with cyanogen bromide following previous protocols (26), then incubated with PLL at 10 mg/mL in 0.1 M NaHCO_3_ overnight, followed by treatment with 50 mM ethanolamine in 0.1 M NaHCO_3_ for 1 h. All these reactions were performed at room temperature with gentle agitation and appropriated washing steps were performed after each step. Finally, BL conjugation was performed by incubation with freshly prepared solution of BP or AX (10 mg/mL) in 0.05 M Na_2_CO_3_/NaHCO_3_ aqueous buffer at pH 10.2 at room temperature with gentle agitation for 24 h, then extra BL solution was added and incubated for additional 24 h at 37°C.

###### 2.2.3.2.2 RAST Protocol

Briefly, 30 µL of subject sera was added over each disc (one functionalized with the corresponding BL-PLL and another blank one only functionalized with PLL without the drug) in a tube and incubated for 3 h with gentle agitation in a wet chamber. After washing, a ^125^I-anti-IgE solution (a pool of antihuman IgE antibodies kindly provided by Thermo Fisher Scientific and radiolabeled in our laboratory) (36) was then added and incubated overnight. Finally, the discs were washed, and radioactivity measured in a Multi Crystal Gamma Counter LB 2111 (Berthold Technologies, Wildbad, Germany). Each assay was performed in duplicate and average values were used for calculating the % RAST result, as a percentage of the maximum of the count per minute (cpm), according to the following equation.


$$\%RAST=\frac{cpm\;(drug\;solid\;phase)-cpm(blank\;solid\;phase)}{cpm\;maximum}$$

Results were considered positive over corresponding cutoffs calculated according to Receiver Operating Characteristic (ROC) curves.

##### 2.2.3.3 RAST Protocol Using DeAn@SiO_2_ NPs

RAST protocol was adapted for using the developed particles as solid phase. Different suspensions of particles in PBS were prepared just before the assay and sonicated: DeAXO@SiO_2_, DeBPO@SiO_2_, DeAXO-BPO@SiO_2_ (as BL-functionalized particles), and De@SiO_2_ (as blank) at optimal concentration of 100 mg/mL. Serum (30 µL) was added over each particle (one functionalized with the corresponding BL-Dendrimer and another blank one only functionalized with dendrimer without the drug) in a tube and incubated overnight with gentle agitation in a wet chamber. After washing, the ^125^I-anti-IgE solution (36) was then added for further incubation overnight with gentle agitation in a wet chamber. Finally, the particles were conveniently washed. All the washing steps used PBS buffer (2 mL x 3 times) involving vortex stirring, centrifugation at RT (5500 rpm, 10 min) and decantation of test tubes by turning over the test tube rack. Radioactivity was measured in a Multi Crystal Gamma Counter LB 2111 (Berthold Technologies, Wildbad, Germany). Each assay was performed in duplicate and average values were used for calculating the % RAST result, as a percentage of the maximum of the cpm, according to the previous equation.

##### 2.2.3.4 Statistical Analysis

Receiver operating characteristic curve (ROC) analysis was performed to determine the best cutoff threshold to obtain the best sensitivity/specificity balance for customized solid phases. RAST results were considered positive over corresponding cutoffs calculated according to ROC curves.

Quantitative variables without a normal distribution (RAST values) for each solid phase were expressed as median, and comparisons between groups of subjects (including tolerant controls, Group A and Group B) were performed using non-parametric analysis for non-related samples, by Kruskall-Wallis and Mann-Whitney tests. Statistically significant differences were considered when p<0.05.

## 3 Results

### 3.1 DeAn@SiO_2_ Preparation and Characterization

SiO_2_ particles were prepared following the well-established Stöber methodology (31, 37). The particles obtained were analyzed by DLS and TEM. DLS result indicates a quite monodisperse sample (**Figure S1**), while microscopy images showed monodisperse spheres of approximately 500 nm of diameter (**Figure S2**).

**Scheme 1:**
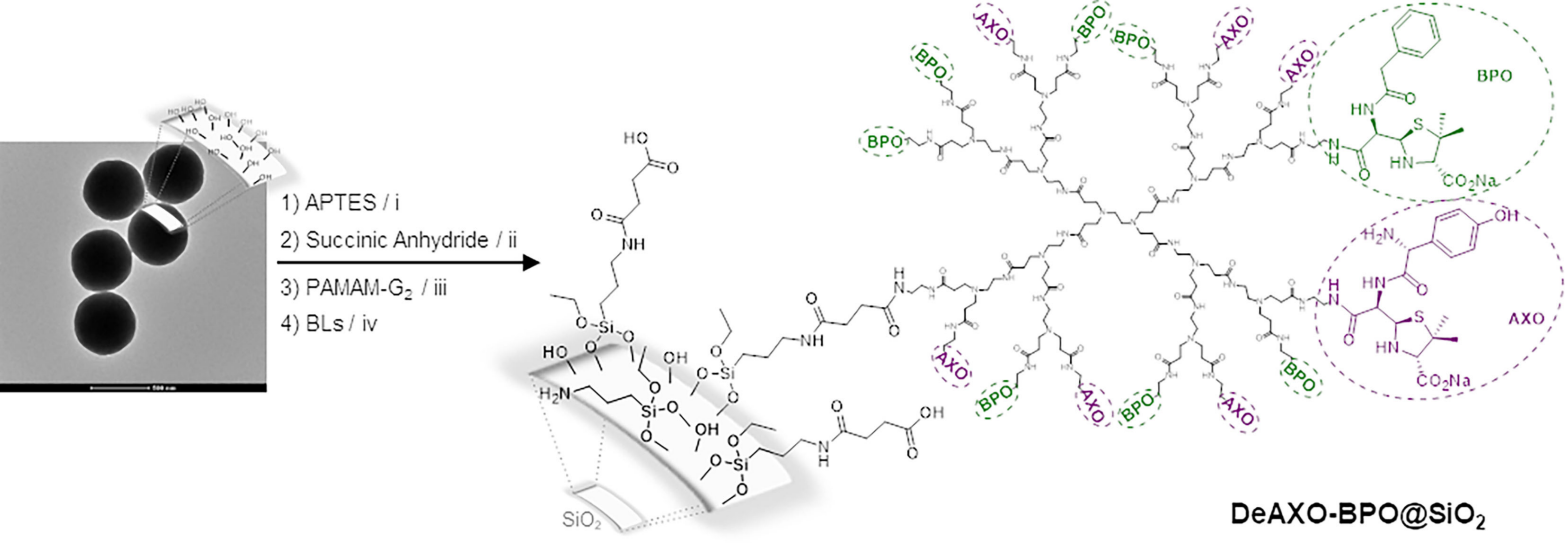
Preparation of DeAn@SiO_2_. (i) Toluene, reflux, 12h; (ii) TEA, DMF, r.t., 12h; (iii) EDCI, NHS, DMF, r.t., 12h; (iv) Na_2_CO_3_/NaHCO_3_ aqueous buffer, 4°C, 7 days. BLs correspond to amoxicillin to obtain DeAXO@SiO_2_ , Benzylpenicillin to obtain DeBPO@SiO_2_ or a 1:1 mixture of amoxicillin:benzylpenicillin to obtain DeAXO-BPO@SiO_2_.

The surface modification of the particles was carried out following our previously described procedure (27), in which the covalent binding between the dendrimer and the silica particle was achieved using APTES and succinic anhydride as linkers (**Scheme 1**). In this sense, particles were reacted with a large excess of APTES overnight to obtain SiO_2_-NH_2_ particles. After the insertion of amino groups, the particles were reacted with succinic anhydride to attach the carboxylic moiety obtaining SiO_2_-CO_2_H. A large excess of the anhydride was also used to encourage the maximum possible amount of amino groups to react, thus introducing a great number of carboxylic units in the particles surface. Generation-2 PAMAM dendrimers were covalently anchored to SiO_2_-CO_2_H particles through the formation of an amide bond with the carbodiimide-activated carboxylic units, obtaining SiO_2_-De. The last step in the preparation of the DeAn@SiO_2_ composites was the reaction of the free amino groups of the dendrimers periphery with the selected BLs. For this study, three different composites were prepared using AX and BP as BLs, either alone or combined into the same particle. In the first case, AX was anchored to the particles, obtaining DeAXO@SiO_2_. When SiO_2_-De particles were reacted with BP, DeBPO@SiO_2_ composites were obtained. For the third kind of particles, both BLs were reacted with the dendrimer modified particles, thus obtaining the DeAXO-BPO@SiO_2_ composites, where both antibiotics resulted arbitrarily anchored to the dendrimer periphery and consequently distributed randomly along the particle surface.

Different techniques were used to monitor the chemical modification carried out in the particles surface. TEM images were recorded to all particles to ensure that no aggregation occurs during the modification process (**Figure S3**). Zeta potential (ξ) measurements were also used since the charge of the particles was expected to change along with the different chemical reactions. As expected, the ξ values (**Table 2**) confirmed the chemical modification of the particle surface. The amount of free amino groups constitutes another factor that undergoes variations throughout the synthetic procedure. These values can be used as an estimation of the degree of functionalization of the particles. In this sense, quantification of amino groups was carried out using a ninhydrin test. As it can be seen in **Table 2**, these values indicate the introduction of 113 µmol of –NH_2_ groups per gram of SiO_2_-NH_2_ particles and 170 µmol of –NH_2_ groups per gram of SiO_2_-De particles. IR spectra have been recorded for all particles. Remarkably, two bands around 1640 and 1550 cm^-1^ appeared in the case of SiO_2_-De, DeAXO@SiO_2,_ DeBPO@SiO_2,_ and DeAXO-BPO@SiO_2_, which were all the particles in which the dendrimer was inserted. These bands, typical for amides groups, confirm the presence of the dendrimer in the particles surface.

**Table 2:** Ninhydrin test and Zeta potential of the functionalized silica particles.

	SiO_2_	SiO_2_-NH_2_	SiO_2_-COOH	SiO_2_-De	SiO_2_DeAXO	SiO_2_DeBPO	SiO_2_DeAXO-BPO
**ξ (mV)**	-64 ± 8	20 ± 5	-40 ± 6	41 ± 7	-8.6 ± 6	-25 ± 5	-12 ± 4
**µmol -NH_2_/g**	–	113	57	170			

Z-potential measurements were carried out in PBS solutions.

**Figure 1:**
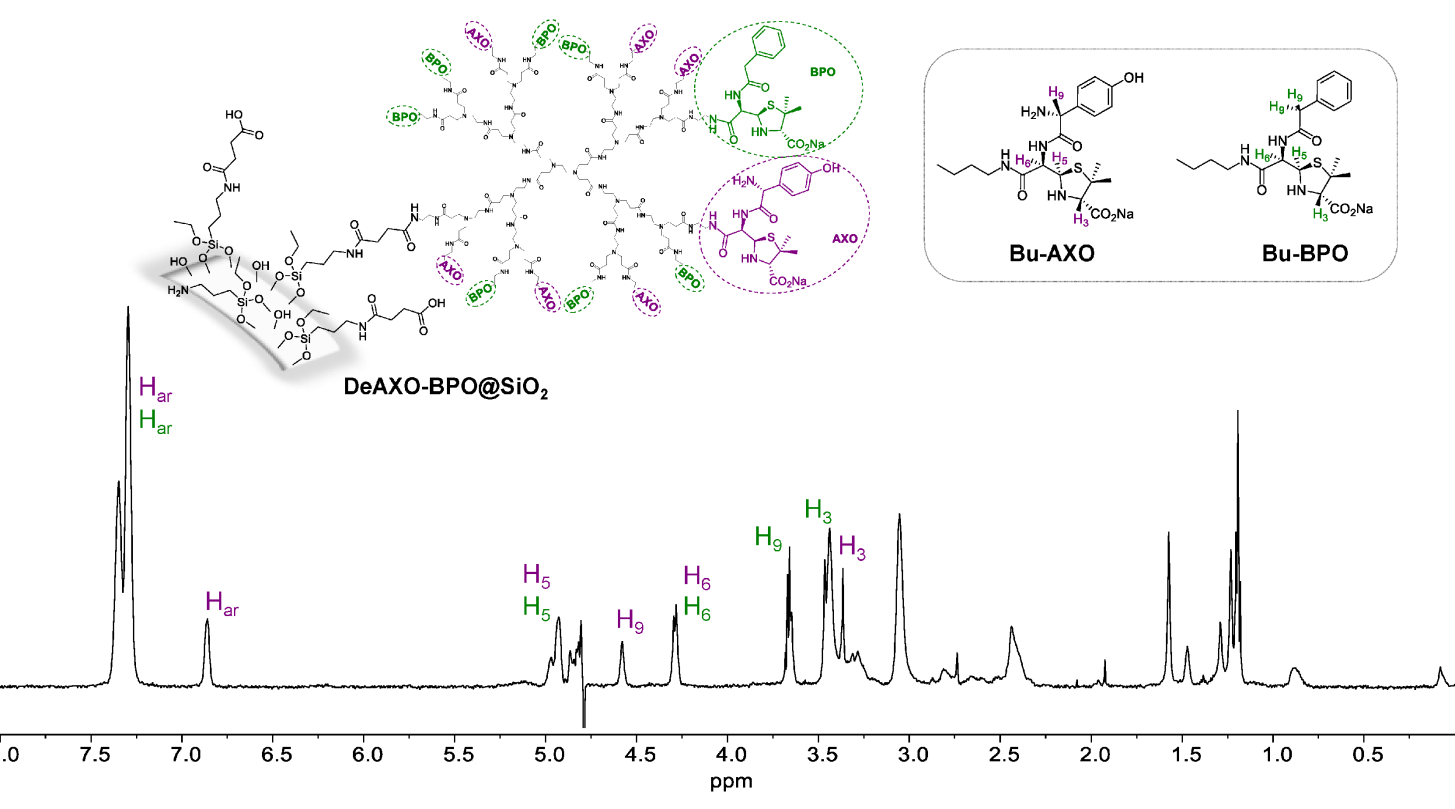
^1^H-NMR spectra of DeAXO-BPO@SiO_2_ composite (suspension in D_2_O). Inset, structures of butylamine-amoxicillin (Bu-AXO) and butylamine-benzylpenicillin (Bu-BPO) where the β-lactam ring is in its open form.

^1^H-NMR spectra of all DeAn@SiO_2_ were recorded (**Figure 1**). Spectra show signals of open β–lactam rings, confirming the effective binding of the corresponding BL to the dendrimers.

### 3.2 Evaluation of Novel DeAn@SiO_2_ Applied to Drug-sIgE Determination and Comparison With Conventional Immunoassay Techniques

After studies regarding the optimal DeAXO@SiO_2_ concentration, we observed with 2 mg of NPs suspended in 20 µL of PBS the highest % of RAST for both pools of patients sera, with high and low level of sIgE, whereas all concentrations showed values close to zero for control patients.

Once the immunoassay conditions were optimized, the ability of DeAn@SiO_2_ to capture serum BL-sIgE antibodies in a group of 21 allergic patients and 10 control subjects with tolerance to BLs was evaluated (**Table 3** and **Table S1**). Moreover, BL-sIgE antibodies were also determined by RAST using the conventional cellulose discs, in order to make comparisons (**Table 3**). All allergic patients were confirmed of an immediate allergic reaction to AX, diagnosed by ST positive to AX and the presence of AX-sIgE by ImmunoCAP. The clinical characteristics of the patients and their ImmunoCAP values are shown in **Table 1**. Patients were further classified as selective to AX (12 subjects, Group A) or cross-reactive to BP (9 subjects, Group B), according to ST or sIgE results to BP, determined by ImmunoCAP (negative for Group A and positive for Group B).

**Table 3:** *In vitro* results of determination of drug-sIgE antibodies of the patients diagnosed with an immediate allergic reaction to AX, performing different immunoassays: standardized ImmunoCAP and customized RAST using cellulose discs or silica particles as solid phase.

Pat	ImmunoCAP			% RAST				
				PLL - discs		De@SiO_2_		
	BPO	AXO	TOTAL IgE	BPO	AXO	BPO	AXO	AXO : BPO
**1A**	0	**0.14**	316	0.71	**21.34**	0	**19**	**11**
**2A**	0.07	**0.26**	3571	0.59	3.02	0	**7.3**	**4.3**
**3A**	0.08	**0.18**	35.1	0	**18.65**	1.3	**8.1**	**3.47**
**4A**	**0.45**	**0.35**	349	**2.93**	**5.95**	1.1	**7.3**	**5.4**
**5A**	**0.44**	**3.14**	2442	**5.01**	**16.89**	0	**19**	**7**
**6A**	**4.29**	**3.72**	1183	**17.23**	**26.08**	1	**20**	**9**
**7A**	0.01	**1.82**	2337	**2.32**	**11.24**	1	**20**	**9**
**8A**	0.01	**0.46**	201	0.74	**5.03**	0	**7.7**	**2.4**
**9A**	0.02	**0.1**	423	0	2.8	0	**8.5**	**3.9**
**10A**	0.05	**0.2**	1545	0.34	**4.52**	0	**9.47**	**5**
**11A**	0	**2.04**	105	**12.88**	**18.1**	0	**19**	**8**
**12A**	0.01	**0.1**	41.3	0.67	**4.51**	0	**10.6**	**4.41**
**13B**	**3.3**	**1.95**	1340	0.44	3.45	**11**	**19**	**15**
**14B**	**2.53**	**4.98**	69.1	**18.24**	**26.69**	**4**	**15**	**11**
**15B**	**1.42**	**1.57**	64.8	**6.85**	**10.85**	**12**	**20**	**17**
**16B**	**0.4**	**1.17**	278	1.51	**14.87**	**6**	**19**	**15**
**17B**	**0.36**	**0.25**	505	0.11	**10.61**	**8.7**	**9.42**	**8.8**
**18B**	**0.75**	**0.74**	211	**37**	**51**	**9.5**	**10.1**	**10.5**
**19B**	**0.27**	**0.83**	591	1.18	**5.87**	**6.3**	**9.17**	**6.7**
**20B**	**0.12**	**0.12**	586	**2.52**	2.65	**10.1**	**8.6**	**9.8**
**21B**	**8.09**	**0.8**	1782	**24.55**	**19.78**	**7.6**	**11.8**	**8.97**

Pat, Patient; Allergenic determinants present in the solid phases; AXO, amoxicilloyl; BPO, benzylpenicilloyl. Positive results (according to standardized or calculated cutoffs) are highlighted in bold style.

**Figure 2:**
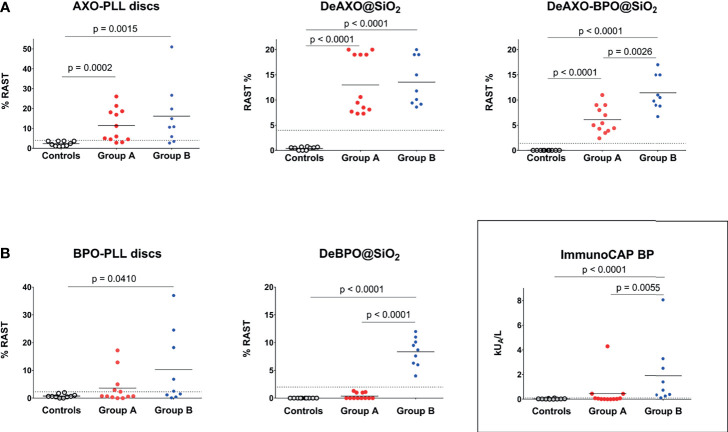
Comparison of different immunoassays fpr BL-sIgE determination. **(A)** AX-sIgE determination using AXO-PLL-discs and DeAXO@SiO_2_ as solid phases in RAST method. Determination of AX-sIgE and BP-sIgE using DeAXO-BPO@SiO_2_ solid phases. **(B)** BP-sIgE determination using the three approaches: ImmunoCAP, BPO-PLL-discs and DeBPO@SiO_2_. Dot plots graphs show individual results for the 3 groups of subjects: tolerant to BLs (controls), AX-selective patients (Group A), and BP-AX cross-reactors (Group B). Positive results are considered over cutoffs (dashed line). Comparisons of quantitative variables without a normal distribution were done by the Mann-Whitney and Kruskall-Wallis tests. Significant differences were considered when p < 0.05, applying Mann-Whitney test for non-related samples.

Results of the sIgE levels are shown in **Figure 2**. We analyzed differences for each solid phase among the three groups (**Figure 2**) by Kruskall-Wallis test. Moreover, analyzing differences for solid phases containing AXO (AXO-PLL discs, DeAXO@SiO_2_, DeAXO-BPO@SiO_2_ in **Figure 2A**) by using Mann-Whitney test, we observed significant high levels in both groups of patients (Groups A and B) compared to control group. Likewise, by using Mann-Whitney test, significant high levels in Group B compared to Group A was observed for DeAXO-BPO@SiO_2_ (p = 0.0026). In the case of solid phases containing only BPO (BPO-PLL discs, DeAXO@SiO_2_ and ImmunoCAP (BP) in **Figure 2B**) significant high levels of BP-sIgE compared to controls were only observed in Group B. Additionally, significant high levels of sIgE were observed in Group B compared to Group A for ImmunoCAP (p = 0.0055) and DeBPO@SiO_2_ (p < 0.0001).

ROC curves for the novel DeAn@SiO_2_ and the conventional BL-PLL-discs were performed to select the cutoffs to obtain the best sensitivity/specificity balance (**Figure S7**). They were established for the corresponding DeAn@SiO_2_ at the following percentages of RAST: ≥ 4% for AX-sIgE using DeAXO@SiO_2_, ≥ 2% for BP-sIgE using DeBPO@SiO_2_ and 1.4% for both AX-sIgE and BP-sIgE using DeAXO-BPO@SiO_2_. Individual data are also represented in **Figure 2A** for AX-sIgE determination and in **Figure 2B** for BP-sIgE quantification. Based on the chosen calculated cutoffs, we observed positive RAST values with DeAXO@SiO_2_ in 21 out of 21 (100%) patients with immediate reactions to AX (Group A plus Group B), with DeBPO@SiO_2_ in 9 out of 9 (100%) patients with cross-reactivity to BP (Group B) and, with DeAXO-BPO@SiO_2_ in 21 out of 21 (100%) included allergic patients to either AX or both BLs (Group A plus Group B). In controls (tolerant subjects to BLs), percentage of RAST was negative in 100% of cases for all three particles (**Table S1**). Cutoffs were established for BL-PLL-discs at the following percentages of RAST: ≥ 4% for AX-sIgE with AXO-PLL-discs and ≥ 2.3% for BP-sIgE with BPO-PLL-discs. According to the selected cutoffs, we observed positive RAST values with AXO-PLL-discs in 10 out of 12 (83%) of patients from Group A and in 7 out of 9 (78%) patients from Group B; therefore, in general positivity was of 80% from all patients with immediate reactions to AX (Group A plus Group B). We observed positive values of BP-sIgE with BPO-PLL-discs in 5 out of 9 (55%) of patients with cross-reactivity to BP (Group B). In controls (tolerant subjects to BLs), RAST was negative in 100% of cases for the two kind of discs (**Table S1**).

**Table 3** shows values of sIgE determination for each BL using the different approaches, taking into account their selected cutoff (standardized for ImmunoCAP and calculated for RAST). The analysis of ImmunoCAP results to AX and/or BP was performed in agreement with the most recently recommended cutoff (≥ 0.1 kUA/L).

The selectivity of the different systems could be studied in patients with no immunological response to BP (**Figure 2B**). Quantification of BP-sIgE in the AX-selective patients, which means false positive results, with the three different methodologies showed that 3 out of 12 (25%) were positive in ImmunoCAP, 5 out of 12 (41%) were positive in RAST with cellulose discs, whereas negative results for all AX-selective patients were obtained with DeBPO@SiO_2_.

## 4 Discussion

Several aspects have to be taken into account for the clinical application of the nanomaterial. Minimizing non-specific protein adsorption interactions between the nanoparticle surface and the sera to be analyzed is one of the most important factors. A versatile surface chemistry that allows a highly density of allergenic molecules in a non-hydrophobic environment has to be undertaken for a correct application of the nanomaterial in the *in vitro* test.

An unquestionable property that nanomaterials provide is the intrinsic high surface area to volume ratio of these materials. This promotes a high functionalization ratio of the particles that can be involved in the presence of a large number of recognition sites in a relatively small amount of the material used. This feature is mostly favorable for overcoming sensitivity limitations. Moreover, due to practical aspects in the manipulation of the particles in the day-to-day hospital work, a particle size optimization is needed since particles had to be easily dispersed in the human sera samples for optimizing molecular interactions (IgE-allergenic determinant and IgE-anti IgE) during incubation steps and easily recovered by centrifugation. To ensure agile, operational, and efficient use of the material in clinical diagnosis, a compromise has been reached by preparing nanocomposites of 500 nm in diameter (27).

Surface chemical modification is a key point when the particles are intended to be used with human samples. In this sense, a previously studied methodology has been used, in which the reproducibility in the composition of the final composites is guaranteed. However, some of the conditions used during the chemical treatment were slightly different from our previous described procedures in order to increase the degree of amino functionalization. One of the advantages of this synthetic methodology is the possibility of checking all reaction steps, by using well known, cheap, and easy procedures.

**Figure 3:**
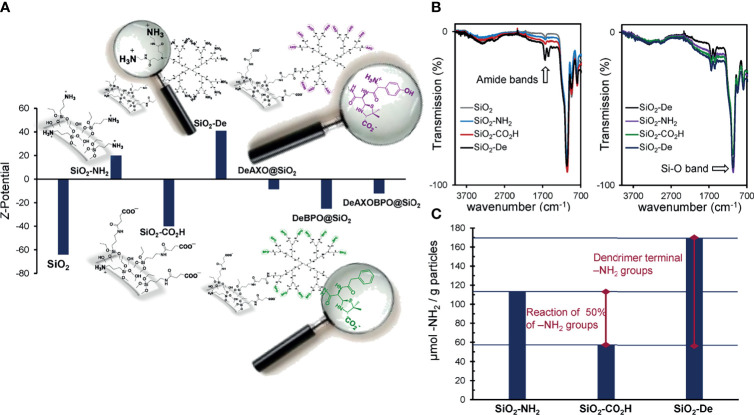
**(A)** Z-potential measurements and **(B)** IR spectra of the functionalized particles; **(C)** quantification of free amino groups of SiO_2_-NH_2_, SiO_2_-CO_2_H and SiO_2_-De.

Zeta potential (ξ) measurements were used to monitor the derivatization process (**Table 2** and **Figure 3**). Naked particles result negatively charged in PBS (solvent that mimics physiological pH 7.4) due to their p*K_a_* (6.8) (27, 38). The introduction of amino groups implies a change of the charge of the SiO_2_-NH_2_ particles surfaces, since in PBS conditions they were protonated (27). A variation in both the value and sing of the charge of SiO_2_-CO_2_H particles surfaces confirms the reaction of a great amount of amino groups, since the introduced carboxylic acid would be in its carboxylate form in PBS (p*K*_a_ ~ 4.0 – 5.0), and hence negatively charge. After reaction with PAMAM-G2 dendrimer, a dramatic change in ξ was also detected for SiO_2_-De particles. A positive charge that duplicates in value the previous measurement was observed. This is consistent with the introduction of 15 amino groups per dendrimer, since generation 2 PAMAM dendrimer possess 16 amino groups and it can be assumed that only one reacted with a carboxylic unit in the particle surface (25). Consequently 15 primary amines were positively charged per dendrimer, while the internal tertiary amines of the dendrimer structure remain non protonated in PBS solutions (39). Obtaining DeAn@SiO_2_ composites involves the reaction of the terminal amino groups of the dendrimers with the carbonyl group on the β–lactam ring of the antibiotics. We can consider that all amino groups on the dendrimer periphery reacted, since we optimized these conditions in our previous work (26). The reaction with the antibiotics caused a shift in the sing of the zeta potential in all cases. For DeAXO@SiO_2_ the amoxicilloyl epitope anchored to the dendrimer possesses a phenol group that must be uncharged in PBS solutions since its p*K*_a_ = 9.6 (and hence should be in its neutral form) (40). However, the carboxyl group must be in its carboxylate form, meaning negatively charged. On the other hand, the primary amino group of AXO has a p*K*_a_ = 7.4, which means that approximately the 50% of these amino groups are protonated at physiological pH (7.4). With this in mind we can assume that 50% of the AXO units anchored to the dendrimers are uncharged (due to the presence of both a negative and a positive charge), while the rest possess a negative charge. In the case of the BPO allergenic determinant, the absence of both the hydroxyl and the amino groups promotes that all BPO units anchored to the dendrimers are negatively charged. This is consistent with the value of ξ observed for DeBPO@SiO_2_. As expected, an intermediated value of ξ is detected for DeAXO-BPO@SiO_2_.

Infrared spectroscopy (IR) has been also used to characterize the particles (**Figure 3B**). As can be observed, the intense band around 1050 cm^-1^, characteristic of Si-O bonds (*ν*_Si-O_), is present in all spectra. Interestingly, two new peaks typical of amide groups (*ν*_C=O_ and *σ*_N-H_ respectively) appear in all particles where the dendrimer is covalently attached.

Ninhydrin test results indicate the introduction of 113 µmol of terminal primary amino groups per gram of particles (**Figure 3** and **Table 1**). When SiO_2_-NH_2_ particles were reacted with an excess of succinic anhydride, an important decrease in the number of terminal amino groups was detected (57 µmol per gram of particles). This implies that approximately 50% of the amino groups in the particles surfaces reacted with succinic anhydride and, as consequence, 56 µmol of carboxylic units have been inserted per gram of SiO_2_-CO_2_H particles (**Figure 3C**). After the covalent coupling with PAMAM-G2 dendrimer, a value of 170 µmol of terminal primary amino groups per gram of SiO_2_-De particles was obtained. Keeping in mind that 57 µmol of amino groups were already present per gram of particles, dendrimers contribute with 113 µmol –NH_2_/g particles. This implies the introduction of 7.5 µmol of PAMAM-G2/g of SiO_2_-De particles (assuming that each PAMAM-G2 possess 15 free amino groups), that is the reaction of approximately 13% of the carboxylic units in the particles (**Figure S4**, ESI). Assuming that particles are prefect spheres of 500 nm of diameter, a density of 2.2 g/cm^3^ (41), and no aggregation occurs during the modification process (**Figure S3**), we can estimate that 1 gram of particles possesses a surface area of approximately 6 m^2^ (see ESI) (27). Therefore, we can estimate the presence of approximately one PAMAM-G2 dendrimer per nm^2^ in SiO_2_-De particles. Taking into account the previous approximations and assumptions, this value seems to indicate an almost complete coverage of the surface of the particles with the dendrimers.

The ^1^H-NMR spectra of DeAXO@SiO_2_, correspond to the one previously published (27). For DeBPO@SiO_2_ (**Figure S5**), we used the ^1^H-NMR spectra of the butylamine-benzylpenicillin conjugate (Bu-BPO) as a model to study the NMR signals (42). In the spectra of DeBPO@SiO_2_ we can clearly observe the presence of the most characteristic peaks of the (3*S*,5*R*,6*R*)-benzylpenicilloate moiety. Signals corresponding to the aromatic ring appear as a multiplet between 7.4 and 7.2 ppm. More interestingly, signals corresponding to H-5 and H-6, bonded to the β–lactam moiety, appear at 4.94 and 4.32 ppm respectively, indicating not only the presence of the BPO unit in the particles, but also the opening of the β–lactam ring of the antibiotics. When this ring is closed, H-5 and H-6 appear closely around 5.5 ppm (33). Additionally, signal corresponding to H-3 appears at 3.50 ppm. All this data indicates the presence of exclusively the open form of the β-lactam ring, which means that all BP present in the particle is covalently attached to the dendrimer, and not adsorbed in the particle surface. In the spectra of DeAXO-BPO@SiO_2_, the presence of both AXO and BPO moieties can be observed. In the aromatic region of the spectra, the signals correspond to the aromatic ring of BPO and AXO (two well defined peaks around 7.34, appearing together with the BPO multiplet, and 6.8 ppm) (23). The absence of any signal around 5.5 ppm indicates that no closed β-lactam rings are present, and for instance, no antibiotics are absorbed to the particle surface. The presence of covalently bound antibiotics in DeAXO-BPO@SiO_2_ is also confirmed by the signals of H-5, H-6 and H-3 of both AXO and BPO units (**Figure 1** and **Figure S6**). The presence of the PAMAM dendrimer was further confirmed in all the spectra by the signal between 3.7 and 2.0 ppm, typical of this kind of dendrimers (23).

After confirming the high density of allergenic determinants of BP and/or AX over the dendrimer grafted particles, their ability to quantify AX-sIgE and/or BP-sIgE was successfully evaluated in a group of well-diagnosed subjects. Comparison of these results with existing methodologies for BL-sIgE determination indicate the great potential of these developed DeAn@SiO_2_ as appropriate platform for capturing antibodies in a specific and selective way.

Nowadays sIgE testing in the context of BL allergy presents relevant limitations in the evaluation of subjects with immediate reactions to penicillins, as the commercial ImmunoCAP method is reported to show low and variable sensitivity (0-50%) (12, 35, 43, 44), although specificity is high (83-100%) (35). The scenario for clinicians is still more complex since not only sIgE detection can fail. Even when the clinical picture is reliable with an immediate drug reaction suggesting IgE-mediated mechanisms, the sensitivity of both ST and serum sIgE detection remains low for BLs (45). Moreover, it is only available for four penicillins (BP, AX, penicillin V, and ampicillin) and one cephalosporin (cefaclor). Therefore, new *in vitro* tools for consistent diagnosis are needed. Conventional RAST using cellulose discs generally show higher sensitivity than ImmunoCAP (43-75%) (8), though it is still suboptimal. This method is not used in routine clinical diagnosis since it can be performed only in specialized laboratories for preparation of disc solid phases and manipulation of radioactive antibodies. To improve the existing techniques, it is necessary to deepen the causes of their limitations or shortcomings, for whose understanding we focus on the characteristics of the solid phases manufactured for the capture of IgE and the context of the peculiarities of drug allergy.

The reasons for the non-favorable detection in the case of drug allergenic molecules include the following: BLs frequently behave as haptens, which require protein binding to become a complete “allergen” with IgE-binding capability. As a consequence, the structure of the drug after covalent binding to the carrier molecule, their available disposition to sIgE interaction, and the nature of the carrier molecule are relevant (17, 19, 45) for detecting the extremely low concentration of sIgE. Another factor contributing to the diagnosis challenge is that some immediate reactions may not be IgE-mediated; although this is important, in this study we did not address this parameter.

AX-sIgE determination using the different solid phases for individual subjects (**Figure 2A**) of Group A shows values of RAST higher for DeAXO@SiO_2_ than for DeAXO-BPO@SiO_2_, as the latter solid phase contains half of the AXO groups, and this must be correlated with the binding; even though results of allergic patients are positive, providing the successful detection of sIgE antibodies to both AX and BP. This could present great usefulness to detect such allergen sIgEs in a first screening for BL allergy diagnosis.

Regarding BP-sIgE determination (**Figure 2B**), the percentage of positive cases was 25% for Group A corresponding to AX selective patients (false-positive results) using ImmunoCAP, in agreement with reported cases of false positive results (15). This could be explained by the presence of a not clinically relevant sIgE to phenylethylamine, a structure that can be present in the ImmunoCAP^®^ and that shares benzyl allergenic epitopes with BP (14). False positive results were also found with BPO-PLL-discs in 45% of patients from Group A, showing suboptimal selectivity. It is interesting that three of the cases of false positive diagnosis to BP (patients 4A, 5A and 6A) coincide with these both methods (**Table 3**). A common characteristic of these patients is the high values of total IgE, in agreement with previous observations indicating a negative influence of levels higher than 200 kU_A_/L in the serum on the detection of BL-sIgE (46). Great results were obtained with DeBPO@SiO_2_ for which all the patients of Group A and Group B were correctly classified (**Figure 2B**).

Although further research is still needed in a higher number of patients for clinical validation, the innovative approach described in this study provides more sensitive and reliable *in vitro* tests with the possibility of testing different BLs, potentially simultaneously. Improvements can be explained by the nano precision, including the following: Monodisperse PAMAM dendrimers offer advantages over PLL polydisperse polymers, displaying controlled and exact number of haptens, resulting in high reproducible materials. The coupling of DeAns on NPs provides solid phases with higher surface area compared to cellulose polymers, which increment the sensibility of the immunoassays given the low concentration of BL-sIgE.

## 5 Conclusions

Nanoscaled DeAn@SiO_2_ materials are the solution for the structural precision that are translated into accurate immunological tests. Mono and bi-epitope DeAn@SiO_2_ composites have been successfully prepared and their ability to recognize sIgE from patient sera has been confirmed. Both, the particle synthesis and its chemical modification protocols have been well established to ensure reproducibility in composition, which is critical for its use in *in vitro* diagnostic applications. The synthetic methodology has been designed in such a way that it allows an exhaustive monitoring of the chemical reactions taking place in the particle surfaces using standard techniques. The control of the degree of functionalization allows the amount of dendrimers covalently attached to be maxima in the prepared nano-composites, ensuring the effective exposure of the hapten-carrier conjugate to specific IgE, and decreasing the uncovered particle surface that could lead to non-specific interactions with sIgE. The three configurations of DeAn@SiO_2_ composites, including the mono-allergenic derivatives and the bi-allergenic DeAXO-BPO@SiO_2_ demonstrated their ability to quantify AX-sIgE and/or BP-sIgE in patients’ sera. These solid phases allowed an excellent distinction between allergic patients and tolerant controls in the reduced selected group of studied subjects, improving the results of existing immunoassays techniques in terms of sensitivity and specificity. Remarkably, these new nano-based platforms provide a suitable tool for accurate sIgE testing in patients to more than one BL simultaneously, representing the starting point for an *in vitro* screening assay that can simplify future diagnostic approaches. Moreover, the described design of DeAn@SiO_2_ can be extrapolated to other penicillins, providing a wider range of culprit BLs to evaluate. All these observations suggest that DeAn@SiO_2_ are potential solid phases for developing diagnostic tools that can overcome limitations of available techniques for BL-sIgE detection.

## Data Availability Statement

The raw data supporting the conclusions of this article will be made available by the authors, without undue reservation.

## Ethics Statement

The studies involving human participants were reviewed and approved by Provincial Ethics Committee of Malaga. The patients/participants provided their written informed consent to participate in this study.

## Author Contributions

EP-I, CM, MT, MM, and YV conceived and designed the study. VG-O performed the Nps synthesis and chemical characterization as well as RAST with particles. CM supervised all *in vitro* tests and analyzed immunoassays data. IJ and MM radiolabeled anti-IgE. IJ and JC performed ImmunoCAP and RAST with discs. ID and MT evaluated and selected patients and controls. YV and MM analyzed all the data, prepared figures, and wrote the paper with input from EP-I, CM, and MT. 3All authors contributed to the article and approved the submitted version.

## Funding

This work was supported by the Spanish Ministerio de Ciencia e Innovación (Proyectos de I+D+I «Programación Conjunta Internacional», EuroNanoMed 2019 (PCI2019-111825-2), Ministerio de Ciencia y Educación (PID2019-104293GB-I00), Instituto de Salud Carlos III (ISCIII) of MINECO (grants cofunded by ERDF: ‘‘Una manera de hacer Europa’’ (Euronanomed Program AC19/00082, PI20/01734, PI18/00095, RETIC ARADYAL, RD16/0006/0001 and RD16/0006/0012, and Miguel Servet II program (CPII20/00028)), Junta de Andalucía and Universidad de Málaga (UMA18-FEDERJA-007), Andalusian Regional Ministry of Health (PE-0172-2018) and Nicolas Monardes Program (RC-0004-2021).

## Conflict of Interest

The authors declare that the research was conducted in the absence of any commercial or financial relationships that could be construed as a potential conflict of interest.

## Publisher’s Note

All claims expressed in this article are solely those of the authors and do not necessarily represent those of their affiliated organizations, or those of the publisher, the editors and the reviewers. Any product that may be evaluated in this article, or claim that may be made by its manufacturer, is not guaranteed or endorsed by the publisher.

## References

[1] Shrivastava V Chauhan PS Tomar RS. Nano-Biomedicine: A Next-Generation Tool for Effective and Safe Therapy. In: Nanobiotechnology. Boca Raton: Apple Academic Press (2020). p. 35–44. doi: 10.1201/9780429292750-3

[2] Nikzamir M Akbarzadeh A Panahi Y. An Overview on Nanoparticles Used in Biomedicine and Their Cytotoxicity. J Drug Deliv Sci Technol (2021) 61:102316. doi: 10.1016/j.jddst.2020.102316

[3] Ladj R Bitar A Eissa M Mugnier Y Le Dantec R Fessi H. Individual Inorganic Nanoparticles: Preparation, Functionalization and In Vitro Biomedical Diagnostic Applications. J Mater Chem B (2013) 1(10):1381–96. doi: 10.1039/c2tb00301e 32260777

[4] De M Ghosh PS Rotello VM. Applications of Nanoparticles in Biology. Adv Mater (2008) 20(22):4225–41. doi: 10.1002/adma.200703183

[5] Fu D Wang Z Tu Y Peng F. Interactions Between Biomedical Micro-/Nano-Motors and the Immune Molecules, Immune Cells, and the Immune System: Challenges and Opportunities. Adv Healthc Mater (2021) 10(7):2001788. doi: 10.1002/adhm.202001788 33506650

[6] Doña I Blanca-López N Torres M García-Campos J García-Núñez I Gómez F. Drug Hypersensitivity Reactions: Response Patterns, Drug Involved, and Temporal Variations in a Large Series of Patients. J Investig Allergol Clin Immunol (2012) 22(5):363–71. 23101312

[7] Salas M Laguna JJ Doña I Barrionuevo E Fernandez-Santamaría R Ariza A. Patients Taking Amoxicillin-Clavulanic can Become Simultaneously Sensitized to Both Drugs. J Allergy Clin Immunol Pract (2017) 5(3):694–702.e3. doi: 10.1016/j.jaip.2017.02.007 28342830

[8] Mayorga C Celik G Rouzaire P Whitaker P Bonadonna P Rodrigues-Cernadas J. *In Vitro* Tests for Drug Hypersensitivity Reactions: An ENDA/EAACI Drug Allergy Interest Group Position Paper. Allergy Eur J Allergy Clin Immunol (2016) 71(8):1103–34. doi: 10.1111/all.12886 26991315

[9] Doña I Romano A Torres MJ. Algorithm for Betalactam Allergy Diagnosis. Allergy (2019) 74(9):1817–9. doi: 10.1111/all.13844 31034613

[10] Mayorga C Ebo DG Lang DM Pichler WJ Sabato V Park MA. Atanaskovic-Markovic, M.; Bonadonna, P.; Jares, E. Controversies in Drug Allergy: *In Vitro* Testing. J Allergy Clin Immunol (2019) 143:56–65. doi: 10.1016/j.jaci.2018.09.022 Mosby Inc. 30573343

[11] Fernandez TD Mayorga C Salas M Barrionuevo E Posadas T Ariza,A. Evolution of Diagnostic Approaches in Betalactam Hypersensitivity. Expert Rev Clin Pharmacol (2017) 10:671–83. doi: 10.1080/17512433.2017.1313110 Taylor and Francis Ltd. 28375040

[12] Torres J Romano A Mayorga C Carmen M Guzman AE Reche M. Diagnostic Evaluation of a Large Group of Patients With Immediate Allergy to Penicillins: The Role of Skin Testing. Allergy (2001) 56(9):850–6. doi: 10.1034/j.1398-9995.2001.00089.x 11551249

[13] Brockow K. Detection of Drug-Specific Immunoglobulin E (Ige) and Acute Mediator Release for the Diagnosis of Immediate Drug Hypersensitivity Reactions. J Immunol Methods (2021) 113101:496. doi: 10.1016/j.jim.2021.113101 34273396

[14] Johansson SGO Adédoyin J Van Hage M Grönneberg R Nopp A. False-Positive Penicillin Immunoassay: An Unnoticed Common Problem. J Allergy Clin Immunol (2013) 132(1):235–7. doi: 10.1016/j.jaci.2012.11.017 23270810

[15] Macy E Goldberg B Poon KYT. Use of Commercial Anti-Penicillin Ige Fluorometric Enzyme Immunoassays to Diagnose Penicillin Allergy. Ann Allergy Asthma Immunol (2010) 105(2):136–41. doi: 10.1016/j.anai.2010.06.014 20674824

[16] Gore A Evans G Rilvën M. Phadia Laboratory Systems. In: Wild D, editor. The Immunoassay Handbook (Fourth Edition). Oxford: Elsevier (2013). p. 617–9.

[17] Doña I Torres MJ Montañez MI Fernández TD. *In Vitro* Diagnostic Testing for Antibiotic Allergy. Allergy Asthma Immunol Res (2017) 9(4):288–98. doi: 10.4168/aair.2017.9.4.288 PMC5446943 28497915

[18] Ariza A Mayorga C Fernández TD Barbero N Martín-Serrano Á. Pérez-Sala D. Hypersensitivity Reactions to SS-Lactams: Relevance of Hapten-Protein Conjugates. J Investig Allergol Clin Immunol Allergol Clin Immunol (2015) 25(1):12–25. 25898690

[19] Ariza A Mayorga C Salas M Donã I Martín-Serrano Á. Pérez-Inestrosa E. The Influence of the Carrier Molecule on Amoxicillin Recognition by Specific Ige in Patients With Immediate Hypersensitivity Reactions to Betalactams. Sci Rep (2016) 6(1):1–10. doi: 10.1038/srep35113 27731424 PMC5059705

[20] Mayorga C Perez-Inestrosa E Molina N Montañez MI. Development of Nanostructures in the Diagnosis of Drug Hypersensitivity Reactions. Curr Opin Allergy Clin Immunol (2016) 16(4):300–7. doi: 10.1097/ACI.0000000000000282 27257940

[21] Mayorga C Perez-Inestrosa E Rojo J Ferrer M Montañez MI. Role of Nanostructures in Allergy: Diagnostics, Treatments and Safety. Allergy (2021) 76:3292–306. doi: 10.1111/all.14764 33559903

[22] Sánchez-Sancho F Pérez-Inestrosa E Suau R Mayorga C Torres MJ Blanca M. Dendrimers as Carrier Protein Mimetics for Ige Antibody Recognition. Synthesis and Characterization of Densely Penicilloylated Dendrimers. Bioconjug Chem (2002) 13(3):647–53. doi: 10.1021/bc0155824 12009957

[23] Montañez MI Najera F Mayorga C Ruiz-Sanchez AJ Vida Y Collado D. Recognition of Multiepitope Dendrimeric Antigens by Human Immunoglobulin E. Nanomed Nanotechnol Biol Med (2015) 11(3):579–88. doi: 10.1016/j.nano.2015.01.006 25661921

[24] Montañez MI Mayorga C Torres MJ Blanca M Perez-Inestrosa E. Methodologies to Anchor Dendrimeric Nanoconjugates to Solid Phase: Toward an Efficient In Vitro Detection of Allergy to β-Lactam Antibiotics. Nanomed Nanotechnol Biol Med (2011) 7(6):682–5. doi: 10.1016/j.nano.2011.07.008 21839054

[25] Ruiz-Sanchez AJ Montañez MI Mayorga C Torres MJ Kehr NS Vida Y. Dendrimer-Modified Solid Supports: Nanostructured Materials With Potential Drug Allergy Diagnostic Applications. Curr Med Chem (2012) 19(29):4942–54. doi: 10.2174/0929867311209024942 22963628

[26] Montañez MI Perez-Inestrosa E Suau R Mayorga C Torres MJ Blanca M. Dendrimerized Cellulose as a Scaffold for Artificial Antigens With Applications in Drug Allergy Diagnosis. Biomacromolecules (2008) 9(5):1461–6. doi: 10.1021/bm701380a 18435560

[27] Vida Y Montañez MI Collado D Najera F Ariza A Blanca M. Dendrimeric Antigen–Silica Particle Composites: An Innovative Approach for Ige Quantification. J Mater Chem B (2013) 1(24):3044–50. doi: 10.1039/C3TB20548G 32261007

[28] Bogas G Mayorga C Martín-Serrano Á. Fernández-Santamaría R Jiménez-Sánchez IM Ariza A. Penicillin and Cephalosporin Cross-Reactivity: Role of Side Chain and Synthetic Cefadroxil Epitopes. Clin Transl Allergy (2020) 10(1):57. doi: 10.1186/s13601-020-00368-1 33292516 PMC7716594

[29] Blanca M Mayorga C Perez E Suau R Juarez C Vega JMM. Determination of Ige Antibodies to the Benzyl Penicilloyl Determinant. A Comparison Between Poly-L-Lysine and Human Serum Albumin as Carriers. J Immunol Methods (1992) 153(1–2):99–105. doi: 10.1016/0022-1759(92)90311-G 1517607

[30] Moore S. Amino Acid Analysis: Aqueous Dimethyl Sulfoxide as Solvent for the Ninhydrin Reaction. J Biol Chem (1968) 243(23):6281–3. doi: 10.1016/s0021-9258(18)94488-1 5723468

[31] Stöber W Fink A Bohn E. Controlled Growth of Monodisperse Silica Spheres in the Micron Size Range. J Colloid Interface Sci (1968) 26(1):62–9. doi: 10.1016/0021-9797(68)90272-5

[32] Romano A Atanaskovic-Markovic M Barbaud A Bircher AJ Brockow K Caubet J. Towards a More Precise Diagnosis of Hypersensitivity to Beta-Lactams — An EAACI Position Paper. Allergy (2020) 75(6):1300–15. doi: 10.1111/all.14122 31749148

[33] Mayorga C Montañez MI Najera F Bogas G Fernandez TD Gil DR. The Role of Benzylpenicilloyl Epimers in Specific Ige Recognition. Front Pharmacol (2021) 12:2021.585890. doi: 10.3389/fphar.2021.585890 PMC7952312 33716734

[34] Brockow K Romano A Blanca M Ring J Pichler W Demoly P. General Considerations for Skin Test Procedures in the Diagnosis of Drug Hypersensitivity. Allergy Eur J Allergy Clin Immunol (2002) 57(1):45–51. doi: 10.1046/j.0105-4538.2001.00001.x-i8 11991289

[35] Fontaine C Mayorga C Bousquet PJ Arnoux B Torres MJ Blanca M. Relevance of the Determination of Serum-Specific Ige Antibodies in the Diagnosis of Immediate β-Lactam Allergy. Allergy Eur J Allergy Clin Immunol (2007) 62(1):47–52. doi: 10.1111/j.1398-9995.2006.01268.x 17156341

[36] Martín-Serrano A Mayorga C Barrionuevo E Pérez N Romano A Moreno E. Design of an Antigenic Determinant of Cefaclor: Chemical Structure–Ige Recognition Relationship. J Allergy Clin Immunol (2020) 145(4):1301–1304.e4. doi: 10.1016/j.jaci.2019.11.036 31821817

[37] Kim JH Kim JS Choi H Lee SM Jun BH Yu KN. Nanoparticle Probes With Surface Enhanced Raman Spectroscopic Tags for Cellular Cancer Targeting. Anal Chem (2006) 78(19):6967–73. doi: 10.1021/ac0607663 17007522

[38] Giri J Diallo MS Simpson AJ Liu Y Goddard WA Kumar R. Interactions of Poly(Amidoamine) Dendrimers With Human Serum Albumin: Binding Constants and Mechanisms. ACS Nano (2011) 5(5):3456–68. doi: 10.1021/nn1021007 21438566

[39] Tanis I Karatasos K. Molecular Dynamics Simulations of Polyamidoamine Dendrimers and Their Complexes With Linear Poly(Ethylene Oxide) at Different PH Conditions: Static Properties and Hydrogen Bonding. Phys Chem Chem Phys (2009) 11(43):10017–28. doi: 10.1039/b913986a 19865754

[40] Rolinson GN. Laboratory Evaluation of Amoxicillin. J Infect Dis (1974) 129(Supplement 2):S139–45. doi: 10.1093/infdis/129.Supplement_2.S139 4601188

[41] Kammler HK Beaucage G Mueller R Pratsinis SE. Structure of Flame-Made Silica Nanoparticles by Ultra-Small-Angle X-Ray Scattering. Langmuir (2004) 20(5):1915–21. doi: 10.1021/la030155v

[42] Montañez MI Najera F Perez-Inestrosa E. NMR Studies and Molecular Dynamic Simulation of Synthetic Dendritic Antigens. Polymers (Basel) (2011) 3(3):1533–53. doi: 10.3390/polym3031533

[43] Blanca M Mayorga C Torres MJ Reche M Moya C Rodriguez JL. Clinical Evaluation of Pharmacia CAP Systemtm RAST FEIA Amoxicilloyl and Benzylpenicilloyl in Patients With Penicillin Allergy. Allergy (2001) 56(9):862–70. doi: 10.1034/j.1398-9995.2001.00995.x 11551251

[44] Sanz ML Gamboa PM Antépara I Uasuf C Vila L Garcia-Avilés C. Flow Cytometric Basophil Activation Test by Detection of CD63 Expression in Patients With Immediate-Type Reactions to Betalactam Antibiotics. Clin Exp Allergy (2002) 32(2):277–86. doi: 10.1046/j.1365-2222.2002.01305.x 11929494

[45] Ansotegui IJ Melioli G Walter Canonica G Caraballo L Villa E Ebisawa M. Ige Allergy Diagnostics and Other Relevant Tests in Allergy, a World Allergy Organization Position Paper. World Allergy Org J (2020) 13:100080. doi: 10.1016/j.waojou.2019.100080 PMC7044795 32128023

[46] Vultaggio A Matucci A Virgili G Rossi O Filì L Parronchi P. Influence of Total Serum Ige Levels on the *In Vitro* Detection of β-Lactams-Specific Ige Antibodies. Clin Exp Allergy (2009) 39(6):838–44. doi: 10.1111/j.1365-2222.2009.03219.x 19400911

