# Supplementary material for: Multiepitope Dendrimeric Antigen-Silica Particle Composites as Nano-Based Platforms for Specific Recognition of IgEs
Source: Front Immunol. 2021 Dec 3;12:750109. doi: 10.3389/fimmu.2021.750109 (PMC8678411; doi:10.3389/fimmu.2021.750109)
Supplement: Supplementary file 1 [file DataSheet_1.pdf]

Supplementary Material

Multiepitope Dendrimeric Antigen-Silica particle composites as nano-based platforms for specific recognition of IgEs

Violeta Gil-Ocaña [1,2,#], Isabel M. Jimenez [3,4,#], Cristobalina Mayorga [2,3,4,#], Inmaculada Doña [3,4], Jose Antonio Céspedes[3], Maria I. Montañez [2,3,˥,*], Yolanda Vida [1,2,˥,*], Maria J. Torres [2,3,4,5,†], Ezequiel Perez-Inestrosa [1,2,†]

[1]Universidad de Málaga-IBIMA, Dpto. Química Orgánica, Campus de Teatinos s/n, 29071 Málaga, Spain

[2]Centro Andaluz de Nanomedicina y Biotecnología-BIONAND. Parque Tecnológico de Andalucía, C/ Severo Ochoa, 35, 29590 Campanillas, Málaga, Spain

[3]Allergy Research Group, Instituto de Investigación Biomédica de Málaga-IBIMA, 29009, Málaga, Spain

[4]Allergy Unit, Hospital Regional Universitario de Málaga, 29009, Málaga, Spain

[5]Universidad de Málaga-IBIMA, Dpto. Medicina, Campus de Teatinos s/n, 29071 Málaga, Spain

[#] Those authors have contributed equally to this work and share first authorship

[˥] Those authors have contributed equally to this work and share senior authorship

[†] Those authors have contributed equally to this work and share last authorship

Table of contents

1.- DSL measurements

2.- TEM images

3.- Estimation of the degree of functionalization of the particles based on the quantification of free primary amino groups

4.- NMR spectra

5.- *In vitro* results of determination of drug-sIgE antibodies of the tolerant subjects to BLs selected in the study

1. DSL measurements

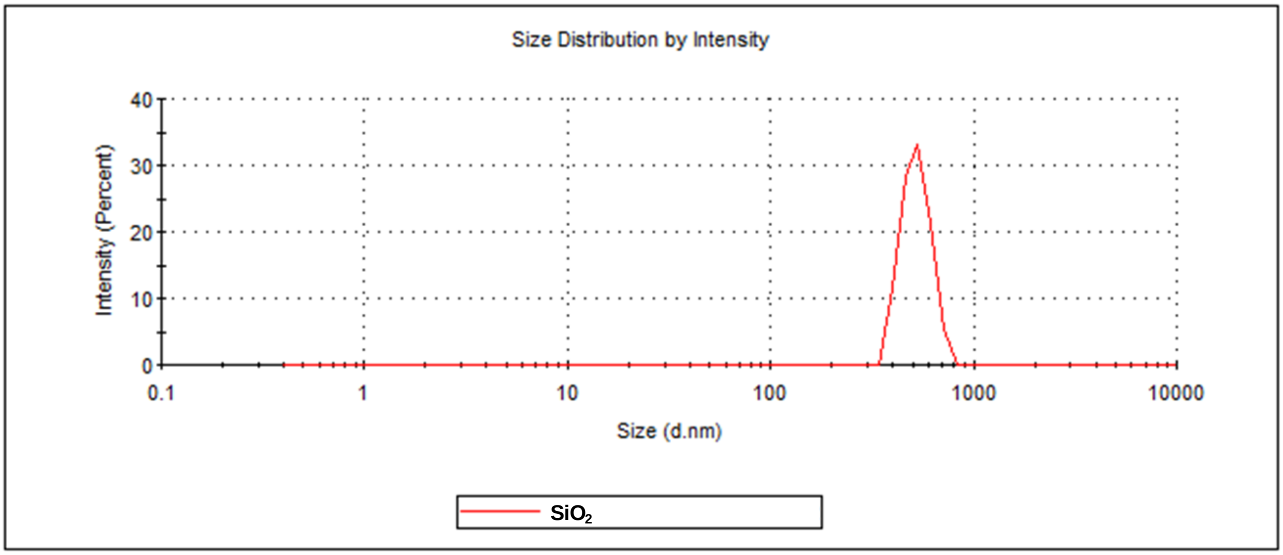

Figure S1. DLS measurements of **SiO_2** particles. Z-average (d) = 536.6 nm; PdI = 0.36.

2. TEM images

Transmission electron microscopy (TEM) images showed monodisperse spheres of approximately 500 nm of diameter.

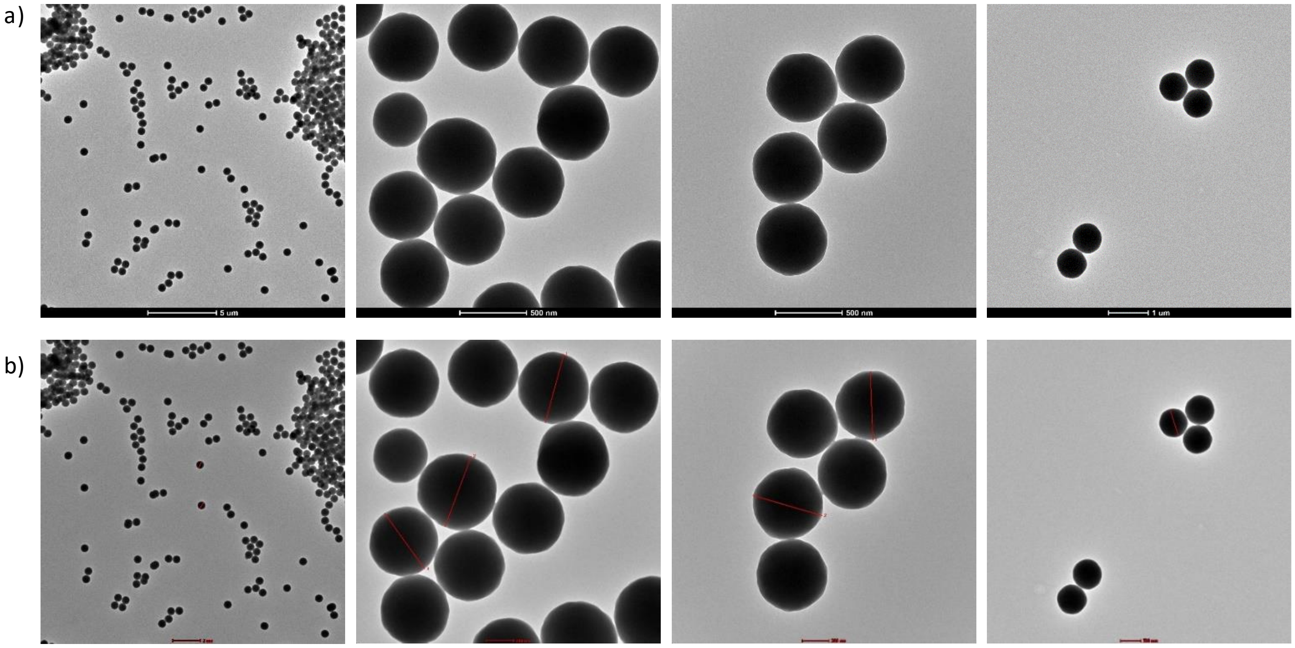

Figure S2. a) TEM images of **SiO_2** particles prepared following the Stöber methodology; b) TEM images treated images with the Thermo Fisher Scientific-TEM Imaging and Analysis program, version 4.71 SP1.

No differences in size and morphology were observed during the surface modification process. We can thus assume that no aggregation occurs during all the functionalization.

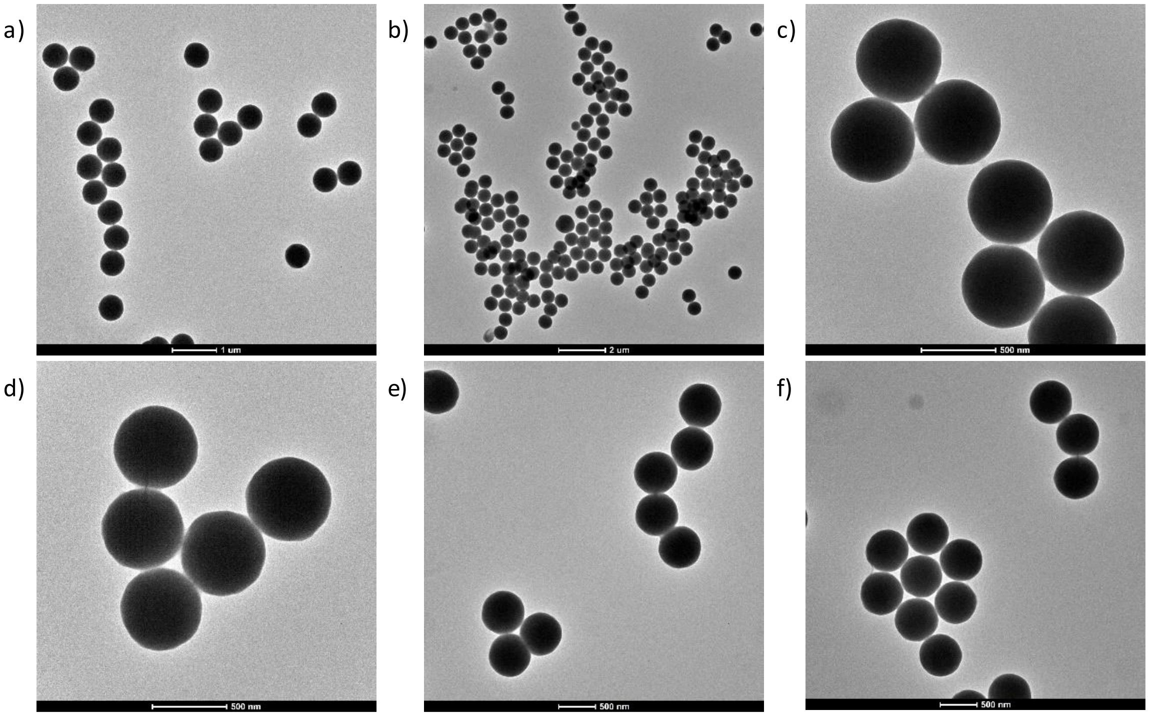

Figure S3. TEM images of a) **SiO_2-NH_2**, b) **SiO_2-CO_2H**, c) **SiO_2-De**, d) **DeAXO@SiO_2**, e) **DeBPO@SiO_2** and c) **DeAXO-BPO@SiO_2**.

3. Estimation of the degree of functionalization of the particles based on the quantification of free primary amino groups.

The quantification of the free primary amino groups present on the particles surface was carried out by using a previously described ninhydrin test procedure. The observed values have been used to estimate the degree of functionalization during the modification procedure.

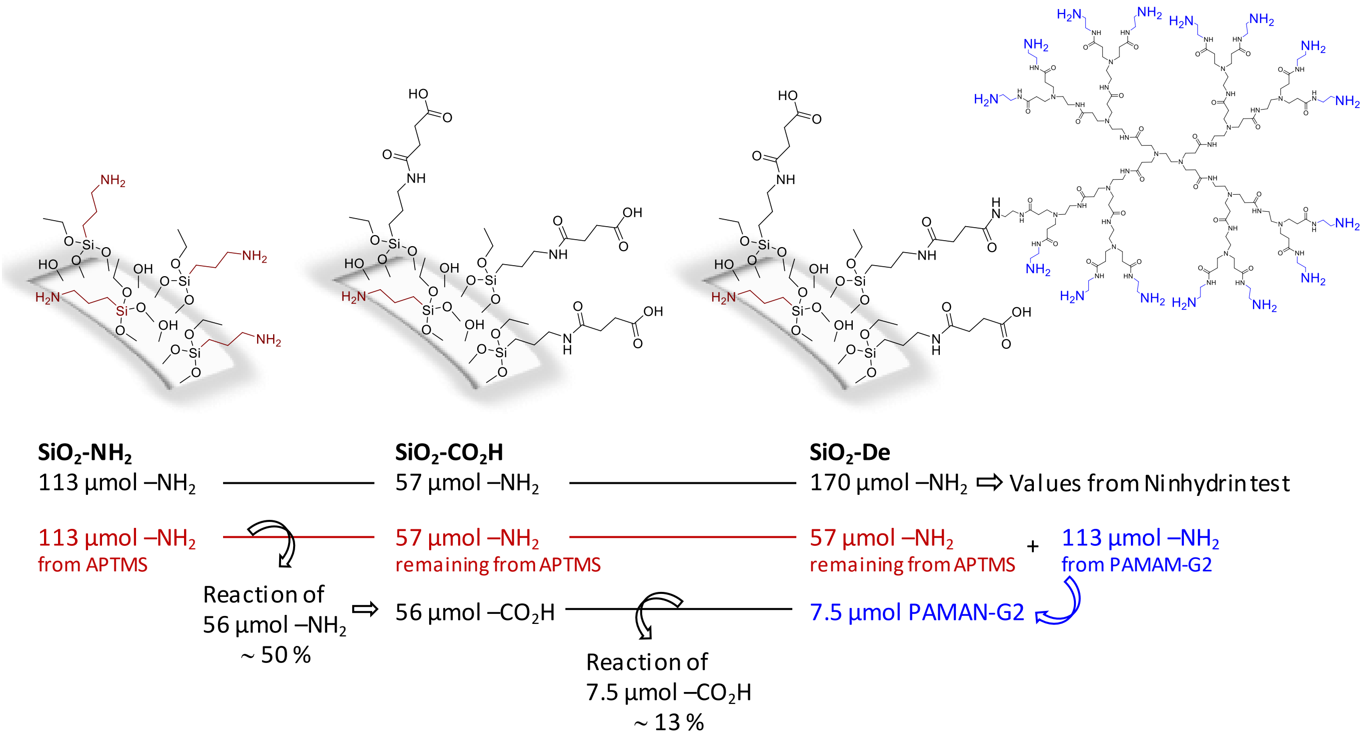

Figure S4. Estimation of the degree of functionalization during the modification procedure based on the values obtained from the Ninhydrin test. All values are referred per gram of particles.

Assuming that the silica particles were perfect spheres of 2.2 g/cm^3 density and 500 nm of diameter, we could estimate:

Density of SiO_2 particles (d): 2.2 g/cm^3

Radius of one sphere (r): 250 nm

Volume of one sphere ($4/3\pi r^3$); $V = 4/3\pi\ (250\ \text{nm})^3 = 65.4\cdot10^6\ \text{nm}^3$

Mass of one sphere (d=M/V): $M = 2.2\cdot10^{-21}\ \text{g/nm}^3 \times 65.4\cdot10^6\ \text{nm}^3 = 1.4\cdot10^{-13}\ \text{g}$

Area of one sphere ($4\pi r^2$); $A = 4\pi\ (250\ \text{nm})^2 = 78.5\cdot10^4\ \text{nm}^2$

Number of spheres per gram of SiO_2 sample:

$1\ \text{g} \times 1\ \text{sphere}/1.4\cdot10^{-13}\ \text{g} = 6.9\cdot10^{12}$ spheres ⇒ $6.9\cdot10^{12}$ spheres/g SiO_2 particles

Surface area per gram of SiO_2:

$78.5\cdot10^4\ \text{nm}^2$/sphere x $6.9\cdot10^{12}$ spheres = $5.5\cdot10^{18}\ \text{nm}^2$ ⇒ $5.5\cdot10^{18}\ \text{nm}^2$/g SiO_2 particles (approximately 6 m^2/g SiO_2 particles)

Ninhydrin test indicates the presence of 170 µmol $–NH_2$ per gram of **SiO_2-De** particles. Of these, 57 µmol $–NH_2$/g corresponds to residual non-reacted amino groups from **SiO_2-CO_2H**. That is, the coupling reaction results in the insertion of 113 µmol $–NH_2$/g from the dendrimers. Assuming that each

PAMAM-G2 dendrimer possess 15 free amino groups after the covalent immobilization, we can estimate the amount of PAMAM-G2 dendrimers per gram of **SiO_2-De** particles:

113 µmol –NH_2 x 1 µmol PAMAM-G2/15 µmol amino groups = 7.5 µmol PAMAM-G2

We can estimate the presence of 7.5 µmol PAMAM-G2/g **SiO_2-De** particles and, for instance:

$7.5\cdot10^{-6}$ mol PAMAM-G2 x $6.023\cdot10^{23}$ PAMAM-G_2 dendrimers/mol PAMAM-G2 = $4.5\cdot10^{18}$ PAMAM-G2 macromolecules

Consequently:

$4.5\cdot10^{18}$ PAMAM-G2 macromolecules/g SiO_2 particles x 1 g SiO_2 particles/$5.5\cdot10^{18}$ nm^2 = 0.82 PAMAM-G2 macromolecules/ nm^2

Approximately 1 PAMAM-G2/nm^2

4. NMR spectra.

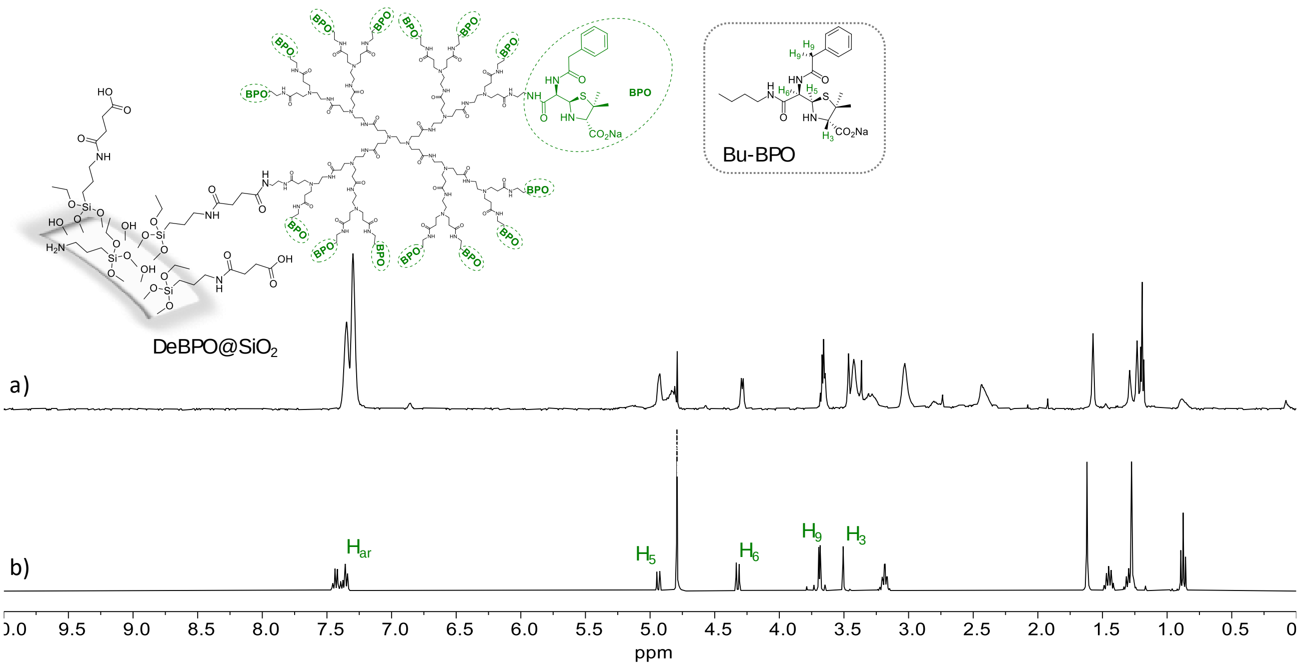

Figure S5. ^{1}H-NMR spectra of a) **DeBPO@SiO_2** composite in D_2O suspension and b) butylamine-benzylpenicilloyl (Bu-BPO) conjugate in D_2O solution.

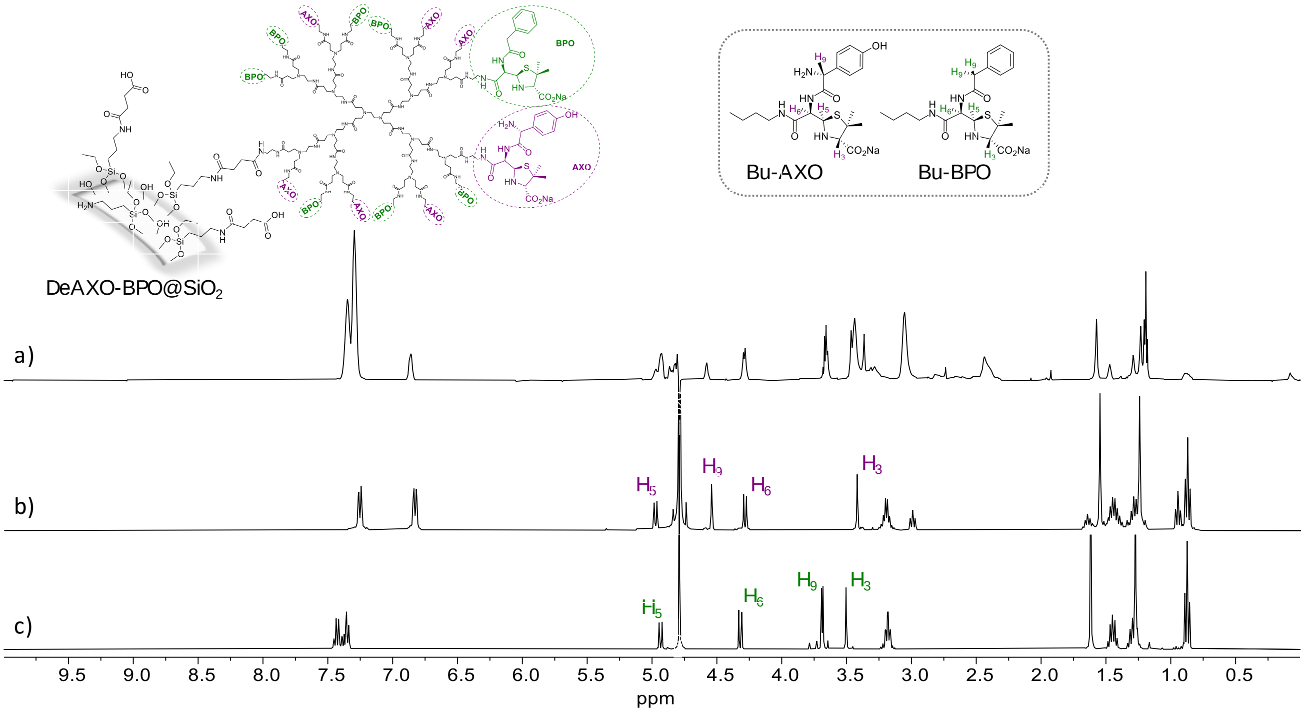

Figure S6. ^{1}H-NMR spectra of a) **DeAXO-BPO@SiO_2** composite in D_2O suspension; b) butylamine-amoxicilloyl (Bu-AXO) conjugate and b) butylamine-benzylpenicilloyl (Bu-BPO) conjugate in D_2O solution.

5. *In vitro* results of determination of drug-sIgE antibodies of the tolerant subjects to BLs selected in the study.

Table S1. ***In vitro*** **results of determination of drug-sIgE antibodies of the tolerant subjects to BLs selected in the study, performing different immunoassays: standardized ImmunoCAP and customized RAST using cellulose discs or silica particles as solid phase.**

Tolerant Subjects	ImmunoCAP			% RAST				
				PLL - discs		De@SiO_2		
	BPO	AXO	TOTAL IgE	BPO	AXO	BPO	AXO	AXO:BPO
**1**	0.03	0.01	7.92	0.63	3.65	0	0.65	0
**2**	0.01	0.01	8.1	0.64	3.68	0	0.54	0
**3**	0.04	0.0005	15	0.47	3.64	0	0.76	0
**4**	0.02	0.06	53.2	0.64	2.37	0	0.52	0
**5**	0	0.04	28.2	0.47	1.32	0	0.46	0
**6**	0.01	0.03	124	0	0.95	0	0	0
**7**	0.02	0.06	5000	2.03	1.94	0	0.44	0
**8**	0	0	1130	0	1.07	0	0	0
**9**	0.06	0.02	5000	1.77	1.09	0	0	0
**10**	0.01	0.01	2000	1.04	3.67	0	0.69	0

Allergenic determinants present in the solid phases: AXO, amoxicilloyl; BPO: benzylpenicilloyl.

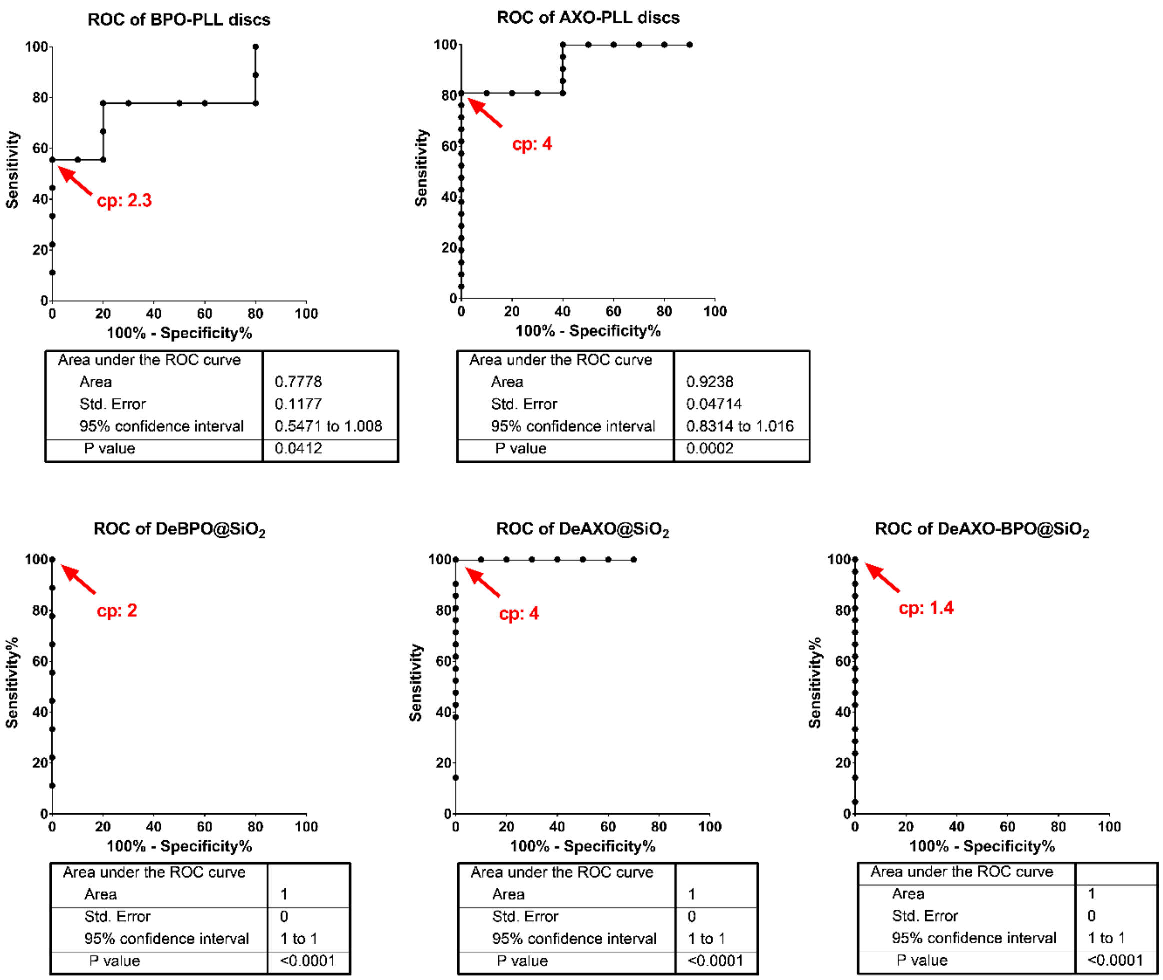

ROC of BPO-PLL discs

Area under the ROC curve	
Area	0.7778
Std. Error	0.1177
95% confidence interval	0.5471 to 1.008
P value	0.0412

ROC of AXO-PLL discs

Area under the ROC curve	
Area	0.9238
Std. Error	0.04714
95% confidence interval	0.8314 to 1.016
P value	0.0002

ROC of DeBPO@SiO_2

Area under the ROC curve	
Area	1
Std. Error	0
95% confidence interval	1 to 1
P value	<0.0001

ROC of DeAXO@SiO_2

Area under the ROC curve	
Area	1
Std. Error	0
95% confidence interval	1 to 1
P value	<0.0001

ROC of DeAXO-BPO@SiO_2

Area under the ROC curve	
Area	1
Std. Error	0
95% confidence interval	1 to 1
P value	<0.0001

Figure S7. ROC curve analysis of each solid phase in RAST method for sIgE determination. Red arrows represent the cutoff points (cp) of percentage of RAST value showing the best sensitivity/specificity balance. *Top:* using **BL-PLL-discs**. *Bottom:* using **DeAn@SiO_2.**
